# Paleo-Rock-Hosted Life on Earth and the Search on Mars: A Review and Strategy for Exploration

**DOI:** 10.1089/ast.2018.1960

**Published:** 2019-10-03

**Authors:** T.C. Onstott, B.L. Ehlmann, H. Sapers, M. Coleman, M. Ivarsson, J.J. Marlow, A. Neubeck, P. Niles

**Affiliations:** ^1^ Department of Geosciences, Princeton University, Princeton, New Jersey, USA.; ^2^ Division of Geological & Planetary Sciences, California Institute of Technology, Pasadena, California, USA.; ^3^ Jet Propulsion Laboratory, California Institute of Technology, Pasadena, California, USA.; ^4^ Department of Earth Sciences, University of Southern California, Los Angeles, California, USA.; ^5^ NASA Astrobiology Institute, Pasadena, California, USA.; ^6^ Department of Biology, University of Southern Denmark, Odense, Denmark.; ^7^ Department of Organismic & Evolutionary Biology, Harvard University, Cambridge, Massachusetts, USA.; ^8^ Department of Earth Sciences, Uppsala University, Uppsala, Sweden.; ^9^ Astromaterials Research and Exploration Science Division, NASA Johnson Space Center, Houston, Texas, USA.

**Keywords:** Subsurface life, Microbial diversity, Biosignatures, Mars, Search for life

## Abstract

Here we review published studies on the abundance and diversity of terrestrial rock-hosted life, the environments it inhabits, the evolution of its metabolisms, and its fossil biomarkers to provide guidance in the search for life on Mars. Key findings are (1) much terrestrial deep subsurface metabolic activity relies on abiotic energy-yielding fluxes and *in situ* abiotic and biotic recycling of metabolic waste products rather than on buried organic products of photosynthesis; (2) subsurface microbial cell concentrations are highest at interfaces with pronounced chemical redox gradients or permeability variations and do not correlate with bulk host rock organic carbon; (3) metabolic pathways for chemolithoautotrophic microorganisms evolved earlier in Earth's history than those of surface-dwelling phototrophic microorganisms; (4) the emergence of the former occurred at a time when Mars was habitable, whereas the emergence of the latter occurred at a time when the martian surface was not continually habitable; (5) the terrestrial rock record has biomarkers of subsurface life at least back hundreds of millions of years and likely to 3.45 Ga with several examples of excellent preservation in rock types that are quite different from those preserving the photosphere-supported biosphere. These findings suggest that rock-hosted life would have been more likely to emerge and be preserved in a martian context. Consequently, we outline a Mars exploration strategy that targets subsurface life and scales spatially, focusing initially on identifying rocks with evidence for groundwater flow and low-temperature mineralization, then identifying redox and permeability interfaces preserved within rock outcrops, and finally focusing on finding minerals associated with redox reactions and associated traces of carbon and diagnostic chemical and isotopic biosignatures. Using this strategy on Earth yields ancient rock-hosted life, preserved in the fossil record and confirmable via a suite of morphologic, organic, mineralogical, and isotopic fingerprints at micrometer scale. We expect an emphasis on rock-hosted life and this scale-dependent strategy to be crucial in the search for life on Mars.

## 1. Introduction

From the mid-1980s to early 1990s, evidence accumulated from both the continental and marine realms of a vast, well-populated underground biosphere that was on par with the total biomass on Earth's surface (Onstott, 2016). The discovery by Stevens and McKinley (1995) of subsurface lithoautotrophic microbial ecosystems (SLiMEs), fueled by H_2_ that was generated by reaction of water with Fe-bearing minerals in basaltic aquifers, had an immediate impact on the planetary science community, especially with respect to the search for extant life on Mars (McKay, 2001). Subsequently, subsurface life on Earth has been discovered at depths of 4–5 km in the continental crust (Moser *et al.,* 2005) and 2.5 km in subseafloor sediments (Inagaki *et al.,* 2015), at temperatures from −54°C to 122°C, at pH values ranging from 3 to 13, and in solutions with ionic strengths up to 7 M for continental crust sites (Magnabosco *et al.,* 2018a). Subsurface life is pervasive on Earth, and rock-based microenvironments offer physical and energetic advantages to their inhabitants compared to the oceans and surface photosphere. In this paper, we refer to “rock-hosted” life, whose existence is critically dependent upon physicochemical processes within the host rock, for example, water-mineral, gaseous, or radiolytic reactions.

The most recent estimate of the mass of Earth's subsurface biosphere is ∼10^30^ cells, which is about 10% that of the surface biosphere (Magnabosco *et al.,* 2018a). One key question when considering the likelihood of finding subsurface life on other planets is how the abundance of Earth's subsurface life may have changed with time, coupled with the evolution and proliferation of surface life, that is, the extensive colonization of land by plant life that began ∼450 million years ago. Some portion of Earth's current global subsurface biosphere is supported directly by or indirectly through thermocatalytic breakdown of organic photosynthate from the surface biosphere while another portion is supported by abiotically produced organic matter or autotrophic carbon fixation. In this paper, we are careful to draw the distinctions between these two types of subsurface ecosystems, focusing on the latter. Over the last decade, it has become apparent that deep subsurface microbial communities are comprised of novel subsurface species with no known closely related surface relatives and that flourish independently of the surface photosphere (Chivian *et al.,* 2008; Osburn *et al.,* 2014; Lau *et al.,* 2016a; Momper *et al.,* 2017), rather than representing the vestiges of transported or buried surface microorganisms struggling to survive on dwindling organic photosynthate (Jannasch *et al.,* 1971). In deep crustal environments, rock-hosted life has been found to comprise entire ecosystems with multiple trophic levels built upon these species (Lau *et al.,* 2016a).

**FIG. 1.**
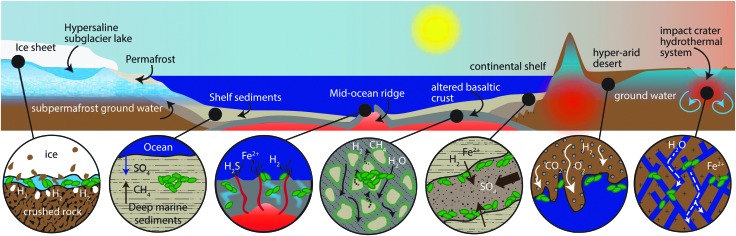
Subsurface biosphere habitats from left to right: Ice and Ice-Rock Interfaces host chemolithotrophs; Marine or Lake Sediments host primarily heterotrophic communities in a high-porosity environment with diffusive flux fueled by organic photosynthate in some places and chemolithotrophic oxidation in others; Ocean Ridges have advective fluids carrying reductants and oxidants, including dissolved gases from magma and water-rock reactions, and abiotic hydrocarbons are oxidized to carbonate mounds (magmatic, non-ridge systems may provide such fluxes on other planets); Deep Basaltic Crust has H_2_-fueled chemolithotrophic communities powered by water-rock reactions; Continental Sedimentary Aquifers are of lower porosity than marine sediments/crust and host mixed heterotrophic and chemolithotrophic communities; and Deep Subsurface Continental aquifers in mafic and siliceous igneous and metamorphic rocks, in some cases fractured by impacts or tectonics, host microorganisms fed by products of radiolysis and water-rock reactions.

Habitats for rock-hosted life may have been—and may still be—present elsewhere in the Solar System (Fig. 1). The sub-ocean silicate crusts of Europa and Enceladus have been proposed to host low-temperature groundwater/hydrothermal systems, leading to chemical/radiolytic reactions, which could supply energy for life (Schulze-Makuch and Irwin, 2002; Hand *et al.,* 2007; McKay *et al.,* 2012, 2008; Pasek and Greenberg, 2012; Vance *et al.,* 2016; Deamer and Damer, 2017; Steel *et al.,* 2017). On Mars, throughout the first 1.5 billion years of its history, surface waters were intermittently present (Fassett and Head, 2011), whereas a more persistent and volumetrically more extensive aqueous environment existed beneath the surface, hosted in crystalline and sedimentary rocks (Clifford and Parker, 2001; Clifford *et al.,* 2010; Des Marais, 2010; Ehlmann *et al.,* 2011; Cockell, 2014a, 2014b), and subsurface brines may still exist today (Orosei *et al.,* 2018). Because of the relative hostility and instability of the martian surface environment—aridity, subfreezing temperatures, frequent climate change due to obliquity cycles, and radiation—compared to Earth or Mars' clement and stable subsurface, sampling rock units that have or may have hosted groundwater warrants top priority in the search for life on Mars.

In this review, we describe a strategy to search for past rock-hosted life on Mars by drawing on the lessons from Earth's record of extant and fossil rock-hosted life. We first describe the environmental history and habitability of Mars. We then review what is currently known about the extent, metabolic diversity, and community structure of present rock-hosted life on Earth, as well as its metabolic products. We next examine the evolutionary history of the enzymes utilized by rock-hosted versus photosynthetic life. We then address how long Earth's rock-hosted life communities, as evident in their biomarkers, have existed and what processes promote preservation of their morphological, mineralogical, isotopic, and chemical traces in the rock record. Finally, we consider the large volumes of rock that constitute past and present habitable environments on Mars and articulate an operational strategy for their exploration for the biosignatures of rock-hosted life.

## 2. The Case for Targeting the Search for Life on Mars to Rock-Hosted Life

On Earth, extensive plate tectonics–driven crustal recycling has removed much of the earliest geologic record and metamorphosed the rest, obscuring the history of the first billion years and extent of the biosphere. On Mars, the ancient geologic record remains largely in place with >50% of the martian rock record from earlier than 3.5 Ga preserved at the surface (*e.g.,* Tanaka *et al.,* 2014), including ancient units uncovered more recently by tectonics, erosion, and impact cratering. As such, if life evolved on Mars contemporaneously with Earth's life, the rocks and biosignatures recording the trajectory of its early evolution are better preserved and more easily accessible than those of time-equivalent periods on Earth.

Over the last decade, *in situ* exploration by rovers and high-resolution mineralogy and stratigraphy by orbiting instruments have revealed the nature of environmental conditions during the first 2 billion years. Globally widespread phyllosilicate minerals (smectites, chlorites, and other hydrated silicates) were formed by aqueous alteration of igneous materials in geologic units from the Pre-Noachian (>4.1 Ga) and Noachian (4.1–3.7 Ga) periods (Mustard *et al.,* 2008; Carter *et al.,* 2013; Ehlmann and Edwards, 2014). The mineral assemblages, chemistry, and geologic setting indicate much of this alteration occurred by water flowing underground (Ehlmann *et al.,* 2011), ranging in depth from shallow sedimentary diagenesis, which depending upon location, comprised acidic, neutral, or alkaline pH fluid (Tosca *et al.,* 2005; Bristow *et al.,* 2015; Yen *et al.,* 2017), to deep, hydrothermal/metamorphic fluid forming serpentine or subgreenschist facies mineral phases, including prehnite and zeolites (Ehlmann *et al.,* 2009, 2011; McSween, 2015). Martian valley networks and open- and closed-basin lake deposits, particularly well preserved during the Late Noachian and Early Hesperian epochs (3.8–3.3 Ga) (Fassett and Head, 2008; Goudge *et al.,* 2016), also record surface water environments. Rover exploration of sedimentary rocks from two different martian basins revealed shallow playas that experienced multiple episodes of diagenesis by acidic waters (Grotzinger *et al.,* 2005; McLennan *et al.,* 2005) and a Hesperian deep lake with multiple later episodes of groundwater diagenesis and/or hydrothermal alteration, possibly as late as the early Amazonian (∼3 Ga) (McLennan *et al.,* 2014; Grotzinger *et al.,* 2015; Martin *et al.,* 2017; Yen *et al.,* 2017; Rapin *et al.,* 2018). Orbital data suggest that other sedimentary basins may have been fed by groundwater (Wray *et al.,* 2011; Michalski *et al.,* 2013), sometimes in communication with magmatic volatiles (Thollot *et al.,* 2012; Ehlmann *et al.,* 2016b). Indeed, surface expressions of impact or volcanic thermal spring systems have been located (Skok *et al.,* 2010; Arvidson *et al.,* 2014; Ruff and Farmer, 2016).

However, after the Late Hesperian (∼3 Ga), evidence for liquid water on Mars is sparse. While even young martian meteorites have evidence for aqueous alteration (*e.g.,* Velbel, 2012), large lava bodies emplaced in the Hesperian and Amazonian do not have hydrated minerals in sufficient abundances to be detectable from orbit (Mustard *et al.,* 2005). Outflow channels, lobate debris aprons, and small valleys occur only near volcanic centers or glacial-like features. Collectively, these data indicate that after a warmer and wetter first ∼1.5 billion years, frozen, arid conditions prevailed over the last ∼3 billion years (*e.g.,* Wordsworth, 2016). Notably, even the Noachian climate may always have been relatively cold and arid (similar to the last 3–3.5 billion years throughout all of Mars' history) with punctuated intervals of higher temperatures due to volcanism (*e.g.,* Johnson *et al.,* 2009; Halevy and Head, 2014), large impacts (Segura *et al.,* 2013; Tornabene *et al.,* 2013), or punctuated release of reduced gases from water-rock reactions (Wordsworth *et al.,* 2017).

If martian life emerged, it is possible that it might have looked like the earliest presently recognized terrestrial record of life, for example, ∼3.4 Ga laminated structures in near-shore, marine facies sediments that are believed to represent anoxygenic photosynthesizing microbial mats (Tice and Lowe, 2004; Tice, 2009) or possible benthic microorganisms in carbonate platforms (Allwood *et al.,* 2009). However, importantly, martian surface water habitats have always been more episodic and extreme than age-equivalent surface habitats on Earth. All evidence suggests that Earth has had an ocean in continuous existence from at least 3.8 Ga and perhaps from as early as 4.4 Ga (Valley *et al.,* 2002). In contrast, the preponderance of the martian geological and mineralogical record along with predictions from climate models suggests that no such body of water on Mars was in continuous existence (Carr and Head, 2015; Wordsworth *et al.,* 2015; Pan *et al.,* 2017). Unlike Earth with its stable axial tilt at 23° ± 1°, Mars' axial tilt fluctuates from 10° to 60° with changes of tens of degrees occurring on timescales of hundreds of thousands of years (Laskar *et al.,* 2004). This has driven episodic reorganization of water reservoirs from the poles to midlatitude belts with concomitant changes in climate cyclically throughout Mars' history (Laskar *et al.,* 2002). Occasional flood events from melting of water ice might have caused outflow channels to debouch in the Northern Lowlands of Mars, forming temporary oceans (Tanaka *et al.,* 2003). Certainly, lakes existed for thousands and perhaps millions of years (Fassett and Head, 2008; Grotzinger *et al.,* 2014, 2015). But by the Noachian-Hesperian boundary (∼3.7 Ga), the atmosphere was <2 bar thick and possibly only tens of millibar thick (Kite *et al.,* 2014, 2017; Edwards and Ehlmann, 2015; Hu *et al.,* 2015; Wordsworth *et al.,* 2015, 2017; Bristow *et al.,* 2017). Mars had also lost much of its protection from solar radiation and galactic cosmic rays by the loss of its dynamo-driven magnetic field at 4.1–3.9 Ga (Acuña *et al.,* 1999) and the subsequent loss of its atmosphere (*e.g.,* Ehlmann *et al.,* 2016a).

Thus, certainly by ∼3.0 Ga, and perhaps earlier, Mars' surface environment had evolved to conditions different from and more challenging to life than the time-equivalent habitats on Earth (Westall *et al.,* 2015). Early martian organisms at the surface would have faced at least seasonally subfreezing temperatures, if not nearly continuous subfreezing conditions with intermittent thaws, surface aridity, and surface radiation doses many times higher than present on early Earth. Ionizing radiation was considerably harsher than that on Earth because of the lack of magnetic field and thin atmosphere (Hassler *et al.,* 2014), and the interaction of UV light with Fe and hydrogen peroxide would have produced photo-Fenton chemistry that is lethal to Earth bacteria (Wadsworth and Cockell, 2017). On the other hand, martian subsurface environments with water were widespread and, comparatively, stable. Evidence for groundwater extends to far more recent martian times than that for surface waters and may still be present today (Orosei *et al.,* 2018). An example is the lake in Gale Crater whose sediments are presently being explored by the Curiosity rover. The lake persisted for up to a few million years (Grotzinger *et al.,* 2015), but the sediments bear markers of sedimentary diagenesis long after the lake had vanished. Crosscutting geologic relationships show that at least several tens of meters of lake sediment had to be eroded, overlain by dunes, the dunes lithified to sandstone, and then crosscut by diagenetic sulfate and silica veins in multiple generations of subsurface fluid flow, persisting even into the Amazonian (Frydenvang *et al.,* 2017; Martin *et al.,* 2017; Rampe *et al.,* 2017; Yen *et al.,* 2017). Fracture networks provided a conduit between habitable subsurface aquifers and more transient surficial habitable systems. Elsewhere, fluid circulation through deep fracture networks driven by hydrothermal activity within impact craters also mobilized fluids from the surface to far beneath the cryosphere (Osinski *et al.,* 2013).

Lastly, an important difference between habitable environments on Earth and Mars may be related to differences in communication between the surface and subsurface. Whereas on Earth warm temperatures and abundant liquid water provided a rapid pathway for recolonizing the surface from the subsurface after impacts (Abramov and Mojzsis, 2009) or global glaciation, on Mars subfreezing surface temperatures and a thick, global permafrost layer (*i.e.,* cryosphere) might have limited communication between surface and subsurface habitats, particularly later in Mars' history (Clifford, 1993; Clifford and Parker, 2001; Harrison and Grimm, 2009; Clifford *et al.,* 2010; Grimm *et al.,* 2017). Therefore, if periodic warm conditions did occur at the surface, the pathways for communication with the subsurface may not have been as easily established for (re)colonization of the martian surface during brief Hesperian surface habitable periods.

Consequently, rock-hosted habitats showing evidence of persistent water warrant considerable attention in the search for martian life (Westall *et al.,* 2015). Some of these systems may have been uninhabitable, perhaps challenged by salinity and acidity (Tosca *et al.,* 2008). Nonetheless, the most globally widespread systems and some sites observed from orbit and explored *in situ* are marked by neutral to alkaline waters of low salinity, which, if on Earth today, would be habitable (Ehlmann *et al.,* 2011; Grotzinger *et al.,* 2015).

Several candidate martian landing sites under consideration for future exploration missions have accessible stratigraphy that may preserve rock-hosted habitats. These include aquifers in volcanic rock and in sedimentary rock. Most immediately, the volcanic rock aquifer with clay minerals, carbonate, and serpentine exposed by erosion at Northeast Syrtis was under consideration for the Mars 2020 rover mission at the time of this submission, and it is accessible in an extended mission from the chosen landing site of Jezero crater. These ancient habitats can and should be explored at high priority as available habitats for martian life, using the lessons and strategies derived from the terrestrial modern and paleorecords of the quantities, nature, and locations of biosignatures of past rock-hosted life.

## 3. Modern Rock-Hosted Life on Earth

### 3.1. Geologic settings with rock-hosted life

On Earth, microbial communities in a wide variety of rock-hosted environments occur globally. Their abundance and community structure reflect physicochemical properties of the rock/water host, the type and rates of energy and nutrient fluxes, geobiological feedbacks, and the geological history of the rock. Though most rock-hosted life is comprised of Archaea and Bacteria, active eukaryotic members exist. These range from protists in deep aquifers (Sinclair and Ghiorse, 1989) to fungi in subseafloor sediment (Orsi *et al.,* 2013; Pachiadaki *et al.,* 2016) and 793 m deep fracture waters in granite (Sohlberg *et al.,* 2015) to multicellular bacteriophagous nematodes (Borgonie *et al.,* 2011). What follows is a brief survey of the variety of rock-hosted ecosystems documented on Earth, some of which could represent terrestrial analogs to potential martian rock-hosted ecosystems (Fig. 1).

The shallowest examples of a rock-hosted ecosystem are highly concentrated cryptoendolithic communities existing millimeters beneath the rock surface, which are not truly “rock-hosted life” as we define the term here (see the introduction). The primary producers of these communities, cyanobacteria and algae, are surface-dwelling photosynthesizing organisms that have retreated to the near subsurface to reduce their exposure to moisture and temperature extremes while retaining access to a sustainable photon flux (Friedmann, 1982; Wong *et al.,* 2010). Some shallow subsurface ecosystems do use their rock/soil hosts for metabolism, meeting our definition of rock-hosted life (Fig. 1). Chemoautotrophic aerobic and anaerobic microorganisms that fix atmospheric CO_2_ reside in barren polar soils and metabolize atmospheric trace gases such as H_2_, CO (Ji *et al.,* 2017), and CH_4_ (Lau *et al.,* 2015; Edwards *et al.,* 2017). Though there is a significant energetic potential for such metabolisms in martian regolith, there is no detectable presence of this metabolism yet (Weiss *et al.,* 2000; Yung *et al.,* 2018).

**FIG. 2.**
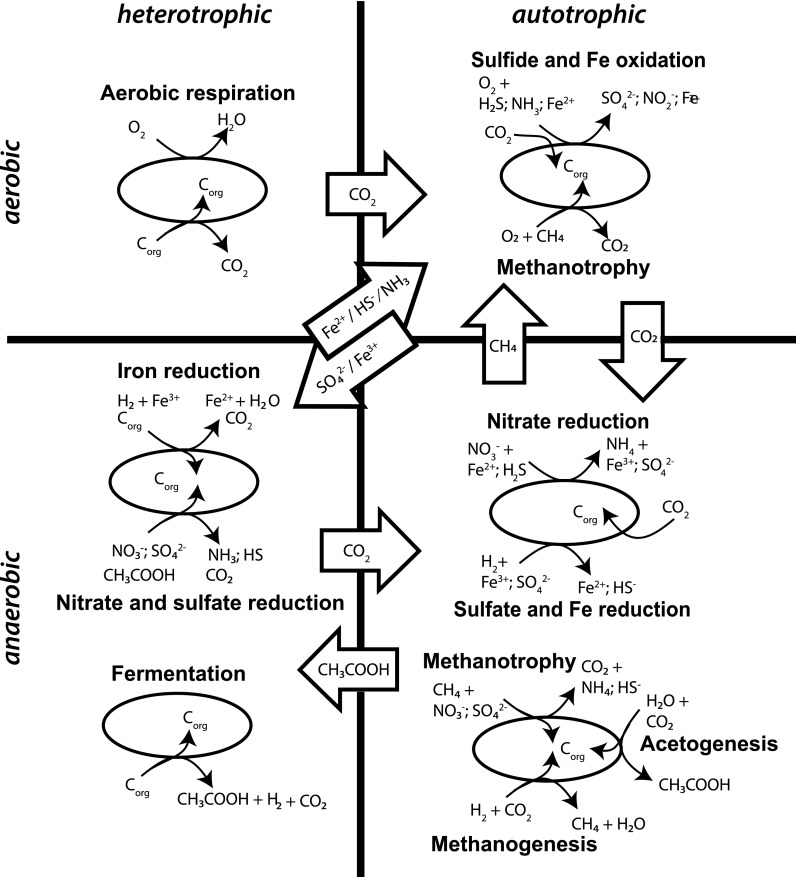
Cartoon of different microbial metabolic processes separated into Aerobic (top), Anaerobic (bottom), Heterotrophic (left), and Autotrophic (right) bins.

For the majority of the continental surface on Earth, heterotrophic bacteria involved in the degradation of organic photosynthate (*e.g.,* cellulose) dominate soil communities (Federle *et al.,* 1986), lacustrine sediments, and shallow aquifers (Balkwill and Ghiorse, 1985). Similarly, organic detritus input from the sea surface, water-column, or continental photosynthates dominate the reductant input to shallow continental margin, subseafloor sediments. Sediment pore waters host a wide variety of specialized microbes that generally use one of a succession of oxidants present in the system (in order of free energy release, O_2_ > Mn oxides > nitrate > Fe oxides > sulfate [Froelich *et al.,* 1979]) to degrade organic matter. The carbon isotope composition of photosynthetic organic matter is distinctive: both terrestrial and marine derived material have a more negative δ^13^C value than marine inorganic carbon. Minerals precipitated in sediment pore space by these microbial metabolic processes have isotopic and chemical compositions that reflect the original organic matter and the oxidant used (*e.g.,* reduced manganese carbonate, rhodochrosite) (Coleman *et al.,* 1982). In organic-rich sediments, degradation processes continue with depth in the pore waters with burial until the organic oxidants are exhausted. In the lowermost oxidation zone, where the least exergonic electron acceptor, CO_2_, remains, methanogenic archaea and acetogenic bacteria largely rely upon abiotic or biotic subsurface H_2_ (Fig. 2). Positive δ^13^C values in carbonates reflect Rayleigh fractionated depletion of CO_2_ by chemolithoautotrophs.

The CH_4_ from methanogenesis may diffuse upward and itself be oxidized anaerobically by archaea, bacteria, and archaeal-bacterial consortia using sulfate or other oxidants in shallow subseafloor sediment (Orphan *et al.,* 2001; Ettwig *et al.,* 2010; Milucka *et al.,* 2012; Haroon *et al.,* 2013; Kits *et al.,* 2015; Cai *et al.,* 2018). Similarly, the acetate from acetogens will be oxidized anaerobically by heterotrophic bacteria (Stevens and McKinley, 1995). At greater depths and temperatures exceeding 100°C, any residual organic photosynthate is thermally matured and may be transformed to oil and/or gas or coal while the host strata become sterilized of indigenous microorganisms, a process described as “paleopasteurization” (Wilhelms *et al.,* 2001). Thus, the microbial communities associated with oil-, gas-, or coal-endowed deposits either represent immigrants arriving with groundwater flow over geological time as the deposits cooled below their maximum temperatures (Tseng *et al.,* 1998), residents indigenous to the sandstone reservoir when oil or gas migrated upward to be trapped (Wilhelms *et al.,* 2001), or contaminants introduced during flooding of or production from the oil reservoirs (Dahle *et al.,* 2008). Although these sediment-hosted microorganisms are living completely or nearly completely off the detritus of photosynthetate, organic matter from abiotic chemosynthetic or biotic chemolithoautotrophic sources would be processed in a similar fashion. The significance of these organic degradation processes is that the inorganic metabolic products may produce characteristic mineral phases, and isotopic and chemical signatures. These can be durable biosignatures that survive for very long periods of time (see Section 4, “Biosignatures of past rock-hosted life”).

In contrast to the environments above where organic photosynthate is important, in continental environments where the water table is hundreds of meters deep (Fig. 1), subsurface communities rely upon chemolithotrophs living off atmospheric or vadose zone gases (Tebo *et al.,* 2015; Jones *et al.,* 2016; Webster *et al.,* 2016), redox reactions with reduced minerals (Mansor *et al.,* 2018), and metals present in carbonate (Barton and Northup, 2007). The chemolithotrophs serve as primary producers for complex communities (Dattagupta *et al.,* 2009; Fraser *et al.,* 2017) and as ecosystem engineers that can excavate large caverns by dissolution of carbonate and deposition of sulfate by the sulfuric acid they produce (Mansor *et al.,* 2018). In water-saturated environments where oxygenated water penetrates deeply into crustal rock such as mountainous terrains of North America (Sahl *et al.,* 2008; Murdoch *et al.,* 2012; Osburn *et al.,* 2014), basaltic flows of the geothermal environments of Iceland (Trias *et al.,* 2017), or taliks in 500 m thick permafrost into underlying Archean metamorphic rock (Onstott *et al.,* 2009), heterogeneous redox conditions create highly exergonic conditions for S, Fe, N, and Mn oxidation; and subsurface microbial communities are dominated by chemolithotrophic primary producers. Where oxygenated seawater comes into contact with marine basaltic crust, chemolithoautotrophs are the primary producers fixing CO_2_ to support substantial biomass by mediating electron transfer at mineralogical redox interfaces from reduced forms of Fe, S, and Mn to the aerobic fluids in pore spaces (Edwards *et al.,* 2012). Unlike the organic-rich sediments discussed above, the geochemical evidence suggests that these rock-hosted communities do not rely upon the groundwater transport of organic photosynthate (Kieft *et al.,* 2018) (Fig. 2).

Of the bioavailable electron donors being utilized by deep, water-saturated, rock-hosted communities, H_2_ is probably a key fuel in the deep biosphere (Nealson *et al.,* 2005), and several abiotic modes of formation exist within rock-hosted environments. In anaerobic volcanic aquifers (Fig. 1), basalt interacts with anaerobic groundwater releasing H_2_, which then supports chemolithotrophic microbial communities by the oxidation of H_2_ at depths of hundreds of meters (Stevens and McKinley, 1995; Mayhew *et al.,* 2013). Even in the absence of O_2_, anaerobic Fe-oxidation via nitrate reduction is a metabolic process that recycles Fe^2+^ produced by Fe^3+^ reduction that can lead to a subsurface Fe-cycle, depending upon the availability of nitrate (Fig. 2; Melton *et al.,* 2014). Given the identification of 70–1100 ppm of nitrate at Gale Crater (Stern *et al.,* 2015) and the Fe-rich nature of the martian crust, the presence of such a metabolic network has been proposed for the martian subsurface (Price *et al.,* 2018).

Radiolysis of groundwater also generates H_2_, H_2_O_2_, and O_2_, that has been shown to sustain subsurface chemolithoautotrophic primary producers by providing not only H_2_ as an electron donor but also electron acceptors, such as sulfate via oxidation of sulfides by radiolytically produced H_2_O_2_ (Lefticariu *et al.,* 2006; Lin *et al.,* 2006; Li *et al.,* 2016). Metagenomic analyses combined with metaproteomic and metatranscriptomic analyses have revealed that within these radiolytically supported communities, a dynamic and temporally varying multi-tier energy pyramid of chemolithoautotrophs exists that recycles biogenic CH_4_ and sulfide and possibly nitrogen, while fixing CO_2_ using the Wood-Ljungdahl pathway and Calvin-Benson-Bassham cycle (Magnabosco *et al.,* 2015, 2018c; Lau *et al.,* 2016b). The bacterial biomass supports multicellular bacteriophagous nematodes at the top of the food chain (Borgonie *et al.,* 2011). These results indicate that radiolysis combined with commensurate syntrophic interactions constantly recharges the redox couplings in these environments; they are not chemically stagnant as claimed by McMahon *et al.* (2018). The radiolytic H_2_ production rate on Mars is just as great as that found in the crustal rocks of Earth despite the lower concentrations of radiogenic isotopes, primarily because of the higher porosity at a given depth due to the lower gravity on Mars (Onstott *et al.,* 2006; Dzaugis *et al.,* 2018; Tarnas *et al.,* 2018).

Cataclastic diminution of silicate minerals in the presence of water also generates H_2_ (Kita *et al.,* 1982) and in the presence of CO_2_ generates CO and O_3_ (Baragiola *et al.,* 2011). H_2_ release during seismic events has been recorded at 3 km depths in South Africa (Lippmann-Pipke *et al.,* 2011), and H_2_ release during rock-crushing at the base on the 3 km thick Greenland ice sheet has been inferred (Telling *et al.,* 2015). The relationship between rock fracturing and/or crushing and subsurface microbial community abundance and activity is not yet resolved and is an avenue of current research in subsurface microbiology. Nonetheless, its implications for subsurface life on Mars, which has fracturing due to impacts and tectonics, have already been proposed (McMahon *et al.,* 2016).

At still greater depths and at temperatures >200°C in peridotite, serpentinization produces abundant H_2_ in high pH fluids (McCollom and Bach, 2009). This H_2_ as well as that generated by radiolysis in turn reacts with transition metal sulfide catalysts to produce CH_4_ and low-molecular-weight hydrocarbons via Fischer-Tropsch-type synthesis (Sherwood Lollar *et al.,* 2002, 2006; McCollom, 2016). The resulting hydrocarbons can either diffuse upward to support chemolithotrophs, methanotrophs, and heterotrophic, alkane-degrading anaerobic bacteria at shallower, cooler temperatures or remain trapped until the host rock has cooled down, whereupon these microbial metabolic clades can penetrate the serpentinite during groundwater flow and utilize the hydrocarbons (Purkamo *et al.,* 2015). These processes support the subsurface microbial communities found in continental ophiolite complexes, such as the Samail Ophiolite (Rempfert *et al.,* 2017) and in metamorphosed komatiites of Archean greenstone belts (Sherwood Lollar *et al.,* 2005). They have also been hypothesized to support a martian subsurface biosphere (Schulte *et al.,* 2006; Westall *et al.,* 2013).

These represent a few examples of the types of rock-hosted microbial communities that are globally distributed across Earth at depths ranging from millimeters to kilometers in a wide range of rock types and that are metabolically and phylogenetically diverse (Mykytczuk *et al.,* 2013). In general, more oxic conditions nearer to the surface yield to more reduced conditions with increasing depth but with important exceptions. Despite the great abundance of organic carbon derived from the surface photosphere in marine sediments and shallow soils, chemolithotrophy is widespread and even dominant in many subsurface environments. This may explain the absence of any correlation of deep subsurface prokaryotic biomass with organic carbon content in the continental subsurface below the soil zone (Magnabosco *et al.,* 2018a).

### 3.2. Fundamental physical and environmental controls on rock-hosted life

The thermal state of the crust constrains the habitable zone. The currently recognized temperature limits for metabolic activity range from −20°C for microorganisms trapped in Siberian permafrost (Rivkina *et al.,* 2000) and −25°C for an aerobic, halophilic heterotroph in laboratory microcosm experiments utilizing ^14^C-labeled acetate (Mykytczuk *et al.,* 2013) up to 122°C for a methanogen isolated from a deep sea vent plume (Takai *et al.,* 2008). Based upon temperature alone, the habitable volume for Earth's continental and oceanic crust has been estimated to be ∼2 × 10^18^ m^3^ (Heberling *et al.,* 2010; Magnabosco *et al.,* 2018a), using global heat flow and surface temperature maps and thermal conductivity estimates. In the case of the continental crust, the average depth to the 122°C isotherm is 4 km; a maximum depth of 16–23 km occurs in the Siberian Craton where mean annual temperatures and heat flow are both lower than average (Magnabosco *et al.,* 2018a). Similar types of calculations for Noachian Mars indicate an average depth to the 122°C isotherm would have been 6–8 km, and the corresponding habitable volume based on temperature constraints alone would also be ∼10^18^ m^3^ (Michalski *et al.,* 2017).

Temperature and possibly the ionic strength of the crustal fluids play a role in constraining the abundance and activity of subsurface life since cell concentrations appear to be inversely correlated with both parameters (Magnabosco *et al.,* 2018a). Organic markers of biodegradation of petroleum suggest that the maximum temperature of the subsurface biosphere may typically be closer to 80–85°C and that salinity >50 g L^−1^ may inhibit low-energy metabolisms such as methanogenesis to even lower temperatures (Head *et al.,* 2014). High concentrations (>220 g L^−1^) of chaotropic salts, such as MgCl_2_, may even preclude life (Hallsworth *et al.,* 2007).

The fluid-bearing (saturated or thin film) porosity that is accessible, that is, with pore throats that are greater than 0.1 μm in diameter, also controls the habitable volume. On Earth, rock strata from 3 to 5 km depth may have a matrix porosity of 0.5% to 1%, but their habitable volume could be as little as 0.05% to 0.002% (Supplementary Fig. S1; Supplementary Information available online at www.liebertonline.com/ast) due to compaction and cementation. On Mars the porosity is likely to be 10% at comparable depths due to the lower gravitational force, and as a result the subsurface habitable volume on Mars may be greater than that of Earth's.

Porosity and permeability also constrain the flux of nutrients and the degree of metabolic activity. For terrestrial life, a finite minimum quantum of energy determined by the reaction ADP + P → ATP must be available through catabolic redox or substrate-level reactions to be metabolically useful (Müller and Hess, 2017). To sustain life, the Gibbs free energy flux (energy per unit time per cell) (Hoehler, 2004; Onstott, 2004) must be equal to or exceed that required for a cell's (Hoehler and Jørgensen, 2013; Onstott *et al.,* 2013) or a syntrophic community's (Scholten and Conrad, 2000) maintenance. Temperature is a principal control on the maintenance energy demand in part because the diffusivity of H^+^ through the cell membranes increases with temperature (van de Vossenberg *et al.,* 1995). As a result, cell metabolic rates must increase with temperature to counteract these effects. The higher the temperature, the higher the nutrient flux needed to maintain a given subsurface biomass. The same may also hold true for salinity as microorganisms need to manufacture internal osmolytes to maintain osmotic pressure and osmolytic production exerts an additional energy requirement (Oren, 1999).

To the extent that higher rock permeability increases groundwater velocities that increase the rate at which reactants flow toward microorganisms hosted by rock strata, higher permeability can lead to a more prolific subsurface biomass by maintaining a nonzero Gibbs free energy (Marlow *et al.,* 2014a). Three other abiotic processes that operate to enhance the local flux of nutrients are chemical gradients or boundaries, physical heterogeneity, and local abiotic and biotic recycling. First, the presence of high electron donor/acceptor chemical spatial gradients in rock units enhances local diffusive fluxes, which leads to higher microbial activity and biomass than in homogeneous units. On modern Earth the most dramatic examples are typically found at the contacts between organic-rich shale and sulfate-bearing sandstone (Krumholz *et al.,* 1997) or oil and water (Bennett *et al.,* 2013) producing higher rates of microbial metabolism than observed some distance away from these contacts. Other examples include fluid-rock redox interfaces at small scales along fractures, detailed below. On Mars an example might be the boundary between a serpentinized olivine-rich unit and an overlying sulfate-rich unit at Northeast Syrtis (Marlow *et al.,* 2014a). Serpentinization of the olivine would generate H_2_ that would then diffuse into the overlying aquifer where it could be microbially oxidized using sulfate, leading to a zone with potential to support high biomass at the boundary. Second, physical heterogeneity can also act to create favorable zones, as is observed in the high cell concentrations within highly fractured and brecciated rock of the Chesapeake Bay impact structure, compared to the overlying marine sediments (Cockell *et al.,* 2012). In this case metabolically active microorganisms are constrained to the fractured rock where they draw down the energy substrates and increase the product concentrations. Though the surrounding massive rock has pore spaces too small for microbes, the diffusive flux of energy substrates from the massive rock into the fractured zone and the diffusion of the products from the fractured zone into the massive rock enhance the biomass residing in the fracture rock (see Section 3.3, Supplementary Fig. S1). Third, abiotic recycling reactions such as radiolysis can continuously generate H_2_ from water and recycle metabolic waste products, such as HS^-^ back into sulfate, and sustain subsurface microbial communities without the need for fluid transport (Lin *et al.,* 2006). “Cryptic sulfur cycling,” in which iron oxides abiotically oxidize sulfide to more oxidized sulfur species, can support organic carbon degradation in non-stoichiometric proportions with a relatively limited sulfur supply (Holmkvist *et al.,* 2011). Finally, syntrophic interactions between different microorganisms also act to sustain subsurface communities by converting waste products back into reactants locally without the need for advective transport (Lau *et al.,* 2016b). Thus, obligately mutualistic metabolism (Morris *et al.,* 2013) may be a characteristic aspect of subsurface microbial communities as a means of avoiding extinctions (Gaidos *et al.,* 1999) because Gibbs free energy will remain nonzero.

### 3.3. Subsurface biomass distribution

**FIG. 3.**
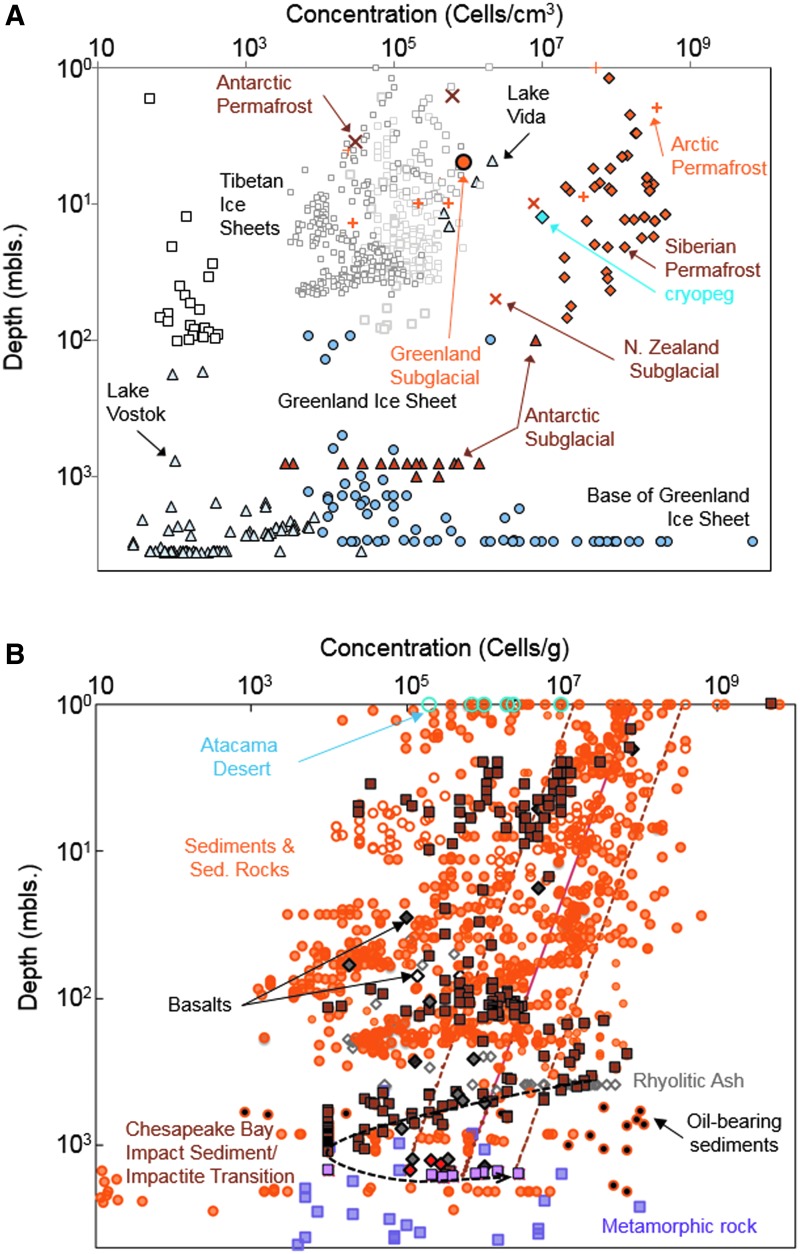
(**A**) Cell concentrations versus depth for ice sheets, subglacial sediments, and permafrost. Open squares = Tibetan glacial ice sheets; brown-filled diamonds = Siberian permafrost; blue-filled diamonds = Siberian cryopeg; light gray-filled triangles = Antarctica ice sheets and lakes; brown-filled triangles = Antarctic subglacial sediments; brown crosses = Antarctic permafrost and subglacial sediment in New Zealand; orange crosses = Canadian High Arctic and Svalbard permafrost; light blue–filled circles = Greenland ice sheet; orange-filled circle = Greenland subglacial sediment. (**B**) Cell concentrations versus depth for rock and soil cores from nonpolar regions. Orange-filled circle = water-saturated sediments or sedimentary rock; orange open circle = vadose zone sediments or sedimentary rock; brown squares = Chesapeake Bay Impact sediments; pink squares = Chesapeake Bay Impact impactite; black-filled orange circle = oil-gas-coal-bearing sediment or sedimentary rock; gray-filled gray diamond = water saturated rhyolitic ash; open gray diamond = deep vadose zone rhyolitic ash; open black diamond = vadose zone basaltic rock; gray-filled black diamond = water-saturated basaltic rock, which includes recent Deccan Trap data from Dutta *et al.* (2018); red-filled diamond = Deccan Trap granite data from Dutta *et al.* (2018); purple square = metamorphic rock. Rest of data are from Magnabosco *et al.* (2018a). Blue open circles = Atacama desert soil from Connon *et al.* (2007) and Lester *et al.* (2007). Solid and dashed lines represent the best-fit power law for subseafloor sediments proposed by Parkes *et al.* (2014).

Magnabosco *et al.* (2018a) recently estimated the total living subsurface prokaryote biomass for Earth was 7–11 × 10^29^ cells of which 2–6 × 10^29^ cells occur in the continental subsurface, 2 × 10^29^ cells exist the oceanic crust, and 3 × 10^29^ cells reside in the subseafloor sediments. Permafrost-affected crust and continental ice sheets cover a large fraction of the continental area and are often considered terrestrial analogs to early Mars. In these, as a function of depth, biomass generally declines; but at all depths, biomass varies by orders of magnitude, depending on the sampling location, including substantial intra-site variation (Fig. 3A). The total cell concentrations for Siberian permafrost sediments can be quite high, ranging from 10^7^ to 10^9^ cells g^−1^ (brown-filled diamonds in Fig. 3A) and diminish with depth up to 100 m and with increasing permafrost age up to 2 Ma (Gilichinsky and Rivkina, 2011). The cell concentrations within the Greenland ice sheet (light blue-filled circles in Fig. 3A) are much lower, on the order of 10^5^ cells cm^−3^, except at the very bottom where the ice sheet is in contact with the Precambrian bedrock and cell concentrations reach 10^9^ cells cm^−3^ as a result of H_2_ generation at the rock-ice interface (Fig. 1). Antarctic permafrost (brown crosses) and subglacial sediments (brown-filled triangles) exhibit cell concentrations that are also greater than those of the adjacent and overlying ice sheets (open triangles). In general, cell concentrations in ice sheets (open squares, triangles, and circles) do not diminish as a function of depth and age as rapidly as observed for permafrost sediments and likely reflect a combination of airborne input flux and *in situ* metabolism (Chen *et al.,* 2016). Cell concentrations are higher near rock-ice interfaces and in dust-rich ice, pointing to the importance of chemical and physical gradients.

Unlike the ice cores, the cell concentrations from rock and sediment cores decline with increasing depth following a power law fit (Fig. 3B). Notable exceptions are the cell concentrations reported for the Chesapeake Bay Impact that increase at a depth of 1.5 km where the highly fractured basement rock exists (brown-filled squares to pink-filled squares in Fig. 3B; Cockell *et al.,* 2012). In soil zones the concentrations range from 10^9^ cells g^−1^ down to as low as 10^5–6^ cells g^−1^ in the case of the Atacama Desert (blue open circles in Fig. 3B; Connon *et al.,* 2007; Lester *et al.,* 2007), considered by some to be a terrestrial analog site for Mars because of its aridity and low organic content. Below 10 m depth, however, cell concentrations do not correlate with water saturation of the pore space (orange open circles versus orange-filled circles in Fig. 3B). For example, the cell concentrations within unsaturated volcanic ash deposits at a depth of 400 m in central Nevada (deep vadose zone) range from 5 × 10^4^ to 5 × 10^7^ cells g^−1^ (gray open diamonds in Fig. 3B; Haldeman and Amy, 1993; Haldeman *et al.,* 1993), which is not significantly different from the cell concentrations reported for water-saturated post-impact sediments of the Chesapeake Bay Impact (brown-filled squares in Fig. 3B; Breuker *et al.,* 2011; Cockell *et al.,* 2012) or Atlantic Coastal Plain Sediments (orange-filled circles in Fig. 3B; Magnabosco *et al.,* 2018a) or Deccan Trap basalts of similar depth (gray-filled diamonds in Fig. 3B; Dutta *et al.,* 2018).

The cell concentrations also do not correlate with the rock type, that is, sedimentary versus igneous versus metamorphic, with the possible exception of salt deposits where cell concentrations are low, ranging from 0.02 to 10^4^ cells g^−1^ (Schubert *et al.,* 2009a, 2009b, 2010; Wang *et al.,* 2016). Despite their paucity within salt, microorganisms exhibit remarkable preservation with viable cells being isolated from salt deposited tens of thousands to hundreds of millions of years in the past (Jaakkola *et al.,* 2016), though the older claims remain controversial (Hebsgaard *et al.,* 2005; Lowenstein *et al.,* 2005).

Cell concentrations on fracture or cavity surfaces are often considerably higher than those of the surrounding fluid or matrix especially if the interface acts to focus redox fluxes. For example, in deep vadose zones cell concentrations up to 10^7^ to 10^8^ cells cm^−2^ and prolific and pigmented biofilms exist on the surfaces of caverns in quartzite (Barton *et al.,* 2014), basalt (Riquelme *et al.,* 2015), and carbonate (Jones *et al.,* 2016). Because of the difficulty of aseptically sampling water-saturated fracture surfaces at depth, only two studies of the cell concentrations on deep fractures have been published. Analysis of modern biofilms, occurring on fracture surfaces in 2.7 Ga metavolcanic rocks at a depth of 2.8 km, revealed 10^5^ cells cm^−2^ with cells occurring in clumps of 2 to >20 (Wanger *et al.,* 2006). Given the fracture width, such a concentration corresponds to a 100× enhancement of the living cell concentration relative to the fracture water. Much lower living cell concentrations, 40 to 2 × 10^3^ cells cm^−2^, have been reported for 186 m deep groundwater-fed fractures in granite (Jägevall *et al.,* 2011). Although these two studies would suggest that deep fracture surfaces do not harbor high biomass concentrations, examination of buried Cretaceous hydrothermal veins reveals preserved organic remains of microbial colonies in mineral surfaces, which by mass would be equivalent to ∼10^9^ cells cm^−2^ (Klein *et al.,* 2015). Similarly, observations of fossil microbial cells in veins in granite indicate a fossil biomass equivalent to 3 × 10^7^ cells cm^−2^ (Pedersen *et al.,* 1997). The higher cell concentrations in fossil biomass versus living biomass result from accumulation of necromass in biofilms on fluid-filled fracture surfaces over time, similar to what is observed in shallow subseafloor sediments (Lomstein *et al.,* 2012).

McMahon *et al.* (2018) stated “the bulk of Earth's massive deep biosphere, and presumably also its fossil record, is a poor analog for any ancient or modern Martian equivalent which, in the absence of a productive surface biosphere, would be much smaller and dominated by chemoautotrophs, not heterotrophs.” This statement is not borne out by existing data. As described in Section 3.1, the cell concentrations in continental rock and groundwater do not exhibit any correlation with dissolved or particulate organic carbon concentrations and are dominantly inhabited by chemolithoautotrophs. This finding contrasts with the observations of shallow subseafloor sediments where cell concentrations do correlate with the organic photosynthate content (Lipp *et al.,* 2008). The deep subsurface environments associated with Phanerozoic-age oil (Head *et al.,* 2014) and coal (Kirk *et al.,* 2015) deposits, where heterotrophic metabolisms would perhaps dominate, comprise only 10^12^ m^3^, or 0.0001%, of the total habitable volume of Earth's subsurface biosphere. The overlap in cell concentrations of the continental rocks with those of deep subseafloor sediments suggests that access to organic photosynthate has little impact on deep subsurface biomass (Fig. 3). The cell abundance data and observed metabolisms do not support the claim that most of Earth's deep biosphere is sustained by heterotrophic metabolism of surface-derived photosynthate. Rather, fracture surface concentrations of 10^5^ to 10^9^ cells cm^2^ are observed in Earth's chemolithoautotrophic communities in settings isolated from organic photosynthate and fueled by chemolithoautotrophy, which is an appropriate analog to Mars. (See Section 4 for a discussion of Earth's fossil record.)

Using simple assumptions from the chemical energy available from basalt weathering (10^−13^ kJ/g-yr), Jakosky and Shock (1998) estimated that over 4 billion years Mars could accumulate 10 g cm^−2^ of biomass from a 100 m thick basalt layer. For comparison, Earth's continental crust is estimated to contain 0.006–0.02 g of extant life cm^−2^, integrated from 1 m depth to the 122°C isotherm (Magnabosco *et al.,* 2018a). The addition of fluid flow from an oxic surface to reducing subsurface, as where sulfate rocks are in contact with serpentinizing rocks, increases this estimate by an additional 10 g of biomass cm^−2^ by enhancing the delivery of reactants and removal products (Marlow *et al.,* 2014a). Calculations of the energy flux from subsurface radiolytic reactions for Mars reveal an energy source comparable to that found on Earth, indicating that the subsurface biomass abundance should be comparable to that of Earth's (Onstott *et al.,* 2006; Dzaugis *et al.,* 2018), and allay the concerns raised about limited oxidant supply to the martian subsurface from the surface (Fisk and Giovannoni, 1999). Radiolysis releases energy into the rock at a rate of 10^−9^ kJ/g-yr based upon the parameters utilized by Onstott *et al.* (2006) of which some fraction is accessible for biomass production depending upon the porosity. This rate is greater than that estimated for weathering reactions and would be even higher on Mars during the Noachian when the radioactive parent isotopes were more abundant. These calculations suggest that adequate energy exists on Mars to support substantial biomass equivalent to that of the rock-hosted biosphere on Earth and that an inhabited Mars would accumulate organic matter over time in subsurface aquifers, completely independent of a habitable martian surface.

### 3.4. Biodiversity and geography

As is the case for biomass, the species richness of deep subsurface environments is highly variable. Subsurface organisms span the branches of the 16S rRNA phylogenetic tree, including deeply rooted lineages (*e.g.,* Methanomada, Archaeoglobi, Korarchaeota, Thermotogae, and Synergistetes) (Magnabosco *et al.,* 2018a). Reports of deep subsurface planktonic communities dominated by a single archaeal (Chapelle *et al.,* 2002) or bacterial (Chivian *et al.,* 2008) species are rare. More commonly, diversity estimates range from over a hundred (Marteinsson *et al.,* 2013) to almost 100,000 (Bomberg *et al.,* 2016) operational taxonomic units or OTUs (at 97% identity in the 16S rRNA gene) within a single fluid sample. To some extent this reflects the improvement in sequencing technology and the fact that the highly variable subregions of the 16S rRNA gene are targeted. It is not unusual to find a large number of OTUs that comprise <1% of the total population (Castelle *et al.,* 2013; Magnabosco *et al.,* 2014), referred to as the rare biosphere (Sogin *et al.,* 2006). Additionally, single-cell genome sequencing of subsurface *Candidatus Desulforudis audaxviator* indicates significant differences in the genomes of single species (Labonté *et al.,* 2015).

Species abundance does not correlate with its ability to influence the overall function of a subsurface community. For example, in continental subsurface environments, methanogens frequently comprise only 2% of the total community, but the primary gas phase is biogenic CH_4_. In the case of one deep subsurface site in South Africa, this biogenic CH_4_ was the principal carbon source for the remainder of the community (Simkus *et al.,* 2015; Lau *et al.,* 2016b). Electron microscopy (Kyle *et al.,* 2008; Middelboe *et al.,* 2011; Engelhardt *et al.,* 2014), single-cell genomes (Labonté *et al.,* 2015), and metatranscriptomic analyses (Lau *et al.,* 2016b) have also revealed that viruses are abundant and actively infecting bacteria and altering their genomes (Paul *et al.,* 2015) in subseafloor sediments and fractured rock aquifers. Because active viral populations can transfer genes between microbial species and control the population density, they may be a vital component of obligately mutualistic metabolic SLiMEs.

One might expect that subsurface diversity would resemble island-like behavior because of the lesser connectedness between subsurface habitats when compared to surface habitats where wind and surface water are transport agents. In island-like ecosystems, the number of species should increase with the size of the island (*i.e.,* volume of groundwater sampled) (Locey and Lennon, 2015). However, Magnabosco *et al.* (2018a) did not find any such correlation. Species richness may instead correlate with greater heterogeneity of microenvironments, which are difficult to characterize in the subsurface. The lack of species richness versus habitat size could also reflect a surprisingly high degree of connectedness between habitats and motility. This is consistent with the presence of the same rare biosphere OTUs in fractures ranging from 0.6 to 3.0 km depth and separated by hundreds of kilometers in South Africa and a general lack of a distance-decay relationship (Magnabosco *et al.,* 2018a). The presence of the same OTUs in fracture water of different isotopic compositions also suggests that these species are actively motile (Magnabosco *et al.,* 2014). The implication of these observations for an early martian subsurface biosphere is that impacts and volcanic activity could have produced zones of sterilized rock, but these zones would have quickly become recolonized by groundwater circulation.

### 3.5. Subsurface metabolic activity

Estimates of the *in situ* metabolic rates of subsurface ecosystems offer insight into their longevity and biomass turnover and provide a better sense of how they impact biogeochemical cycles on a planetary scale, though obtaining accurate estimates has proven challenging (Orcutt *et al.,* 2013). Geochemical estimates of the electron production rate from marine subsurface *in situ* microbial activity range from ∼5 mol e^-^ L^−1^ yr^−1^ at seafloor hydrothermal vents (Wankel *et al.,* 2011) to ∼2 to 100 pmol e^-^ L^−1^ yr^−1^ in oligotrophic subseafloor red clays (Røy *et al.,* 2012) to 0.004 to ∼4 pmol e^-^ L^−1^ yr^−1^ for continental deep fractured rocks and consolidated sediments (Kieft and Phelps, 1997), estimates spanning more than 15 orders of magnitude. Recent metabolism-agnostic approaches utilize isotopic labeling and measurements of the D/L of aspartic acid of bulk subseafloor sediment (Lomstein *et al.,* 2012) and of cells separated from deep continental fracture fluids (Onstott *et al.,* 2013) to determine the bulk rate of growth and repair, often referred to as cell turnover. These analyses yielded cell turnover times ranging from 73,000 years for subseafloor sediments to 1.7–1.8 years for 60°C fracture water and imply that *in situ* metabolic rates are strongly temperature dependent (Xie *et al.,* 2012; Onstott *et al.,* 2013; Trembath-Reichert *et al.,* 2017). In the case of the 60°C fracture water dominated by a single species of autotrophic sulfate-reducing bacterium, the cell turnover time corresponded to a metabolic rate of 2.6–2.8 nM of sulfate per year or 21–22 nmol e^-^ L^−1^ yr^−1^ (Onstott *et al.,* 2013). The shorter cell turnover times observed in deep continental fracture water are also consistent with metatranscriptome and metaproteome observations from similar environments that revealed significant intracommunity recycling of metabolic waste products (Lau *et al.,* 2016b). Recycling of biogenic CH_4_, sulfide, and CO_2_ suggests that initial geochemical approaches for estimating metabolic rates (Phelps *et al.,* 1994) may have underestimated the rates of cell turnover and thus metabolic rates. Despite the challenges posed in accurately estimating the metabolic rates of subsurface microbial ecosystems, recent *in situ* approaches utilizing natural ^14^C (Simkus *et al.,* 2015) suggest that a wide range can be found that correlates with the energy fluxes and temperatures. The longer turnover times in colder ecosystems raise questions about the nature and rate of evolution in subsurface ecosystems, which are also characterized by physicochemical conditions that are more stable over time than surface ecosystems and where microorganisms are exposed to low radiation dosage rates relative to those of surface ecosystems (Teodoro *et al.,* 2018).

### 3.6. Evolution of chemoautotrophic versus photosynthetic pathways

The depiction of subsurface microbial environments above as dominantly suboxic conditions near the surface yielding to more reduced conditions with increasing depth is the result of the evolutionary emergence of oxygenic photosystem II in cyanobacteria. Prior to this emergence, both surface and subsurface habitats were likely dominated by anaerobic metabolisms such as methanogenesis. Recently retrieved genomes of methanogens belonging to Crenarchaeota have revised our understanding of when methanogenic metabolisms evolved. The discovery of Verstraetearchaeota (Vanwonterghem *et al.,* 2016) and Bathyarchaeota (Evans *et al.,* 2015) indicates that the methanogenic metabolic pathway must have arisen after the split between Archaea and Bacteria from the Last Universal Common Ancestor, LUCA, but prior to the split between the Crenarchaeota and Euryarchaeota. This places the time for the emergence of this pathway in the Paleoarchean or Hadean. The youngest bound on age for the development of the methanogenic pathway is ∼3.25 Ga, that is, prior to or during the proposed Archean expansion of genes, based upon molecular clock analyses (David and Alm, 2011), and likely developed in the stem of Archaea (Betts *et al.,* 2018).

Recently sequenced genomes from non-photosynthetic members of the Cyanobacteria phylum indicate that oxygenic photosynthesis arose within Cyanobacteria after the split of photosynthetic Cyanobacteria (now the class of Oxyphotobacteria) from the other non-photosynthetic Cyanobacterial lineages, Melainabacteria and Sericytochromatia (Soo *et al.,* 2017). The age for this divergence has been estimated to be 2.6 to 2.5 Ga based upon molecular clocks (Shih *et al.,* 2017), which lies between the ∼2.8 Ga age for the stem of the Cyanobacteria and the 2.2 Ga age for oxygenic photosynthesis emergence estimated from a different molecular clock approach (Magnabosco *et al.,* 2018b). This is consistent with the theory that photosystem II evolved from a Mn-carbonate-oxidizing enzyme within a suboxic, neutral pH paleoocean (Johnson *et al.,* 2013) to a HCO_3_^−^-oxidizing oxygenic photosystem and then to H_2_O-oxygenic photosynthesis (Dismukes *et al.,* 2000) just prior to the rise of O_2_ during the Great Oxidation Event at *ca.* 2.3 Ga (Betts *et al.,* 2018). During this transition, surface anaerobic ecosystems would have either started going extinct or would have adapted to higher O_2_ levels, perhaps incorporating aerobic metabolic pathways, whereas deep subsurface ecosystems would have remained relatively unaffected.

The molecular clock constraints on the non-oxygenic phototrophic-bearing phyla, Chloroflexi (green nonsulfur bacteria) and Chlorobi (green sulfur bacteria) suggest that the origin of their stems dates from no earlier than ∼3 Ga with Fe^2+^-oxidizing green sulfur bacteria likely being the most ancient (Magnabosco *et al.,* 2018b). The inferred Fe^2+^ phototrophic mat structures in the 3.45 Ga Buck Reef Chert (Tice and Lowe, 2004) predate this stem age for all phototrophic lineages. The remaining phototrophic bacteria within the phyla Proteobacteria and Clostridia likely acquired their abilities by later horizontal gene transfer. The discrepancy in the timing for the emergence of phototrophy between the fossil record, 3.45 Ga, versus that of molecular clock models, 3.0 Ga, requires resolution, but the emergence of oxygenic photosynthesis is much later.

A critical nutrient to the expansion of both subsurface and surface life on any planet is the availability of nitrogen as an aqueous species. On Earth, microorganisms evolved the ability to fix N_2_ into ammonia with the development of nitrogenase to overcome this constraint. Nitrogenases, Nif proteins, are complex enzymes, utilizing iron, molybdenum, and/or vanadium, that exist in both bacterial and archaeal domains. Phylogenetic comparison of genes that comprise nitrogenases and a complement of proteins required for their regulation indicate that nitrogenases emerged in anoxic sulfidic environments on Earth within obligate anaerobic thermophilic methanogens and were transferred to obligate anaerobic clostridia (Boyd *et al.,* 2015), both common subsurface microorganisms. As Nif proteins were adopted first by the aerobic diazotrophic lineage Actinobacteria and then by the more recently evolved aerobic Proteobacterial and Cyanobacterial lineages, the Nif protein suite became more complex to protect the core MoFe-bearing proteins from O_2_ (Boyd *et al.,* 2015). Although it is not clear whether the emergence of the more complex protein occurred prior to or after the Great Oxidation Event, it is certain that the ancestral protein emerged in an anoxic environment when the demands for aqueous nitrogen species exceeded the abiotic supply. The implications for martian ecosystems are that nitrogenase would have also likely emerged within an anaerobic subsurface environment, not in the oxic surface environment.

Experiments on the effects of low pN_2_ on diazotrophic nitrogen-fixing soil bacteria have shown that they could grow in N_2_ partial pressures of 5 mbar but not 1 mbar (Klingler *et al.,* 1989). This result suggests that further experiments on wild-type species are required to determine whether the evolution of pN_2_ in the martian atmosphere was a significant deterrent to the expansion of early life, especially after Mars lost most of its atmosphere. Analyses of the nitrogen budget and of nitrogen cycling from deep subsurface environments in South Africa indicate that the pN_2_ is higher at depth than on the surface, that most of this N_2_ originates from the rock formations through nitrogen cycling, and that N_2_ is being actively fixed in the subsurface by microbial communities (Silver *et al.,* 2012; Lau *et al.,* 2016b). Given the presence of a cryosphere barrier to diffusion on Mars, the nitrogen availability and perhaps even the pN_2_ of subsurface brines are likely to be higher there than on the martian surface.

## 4. Biosignatures of Past Rock-Hosted Life

**FIG. 4.**
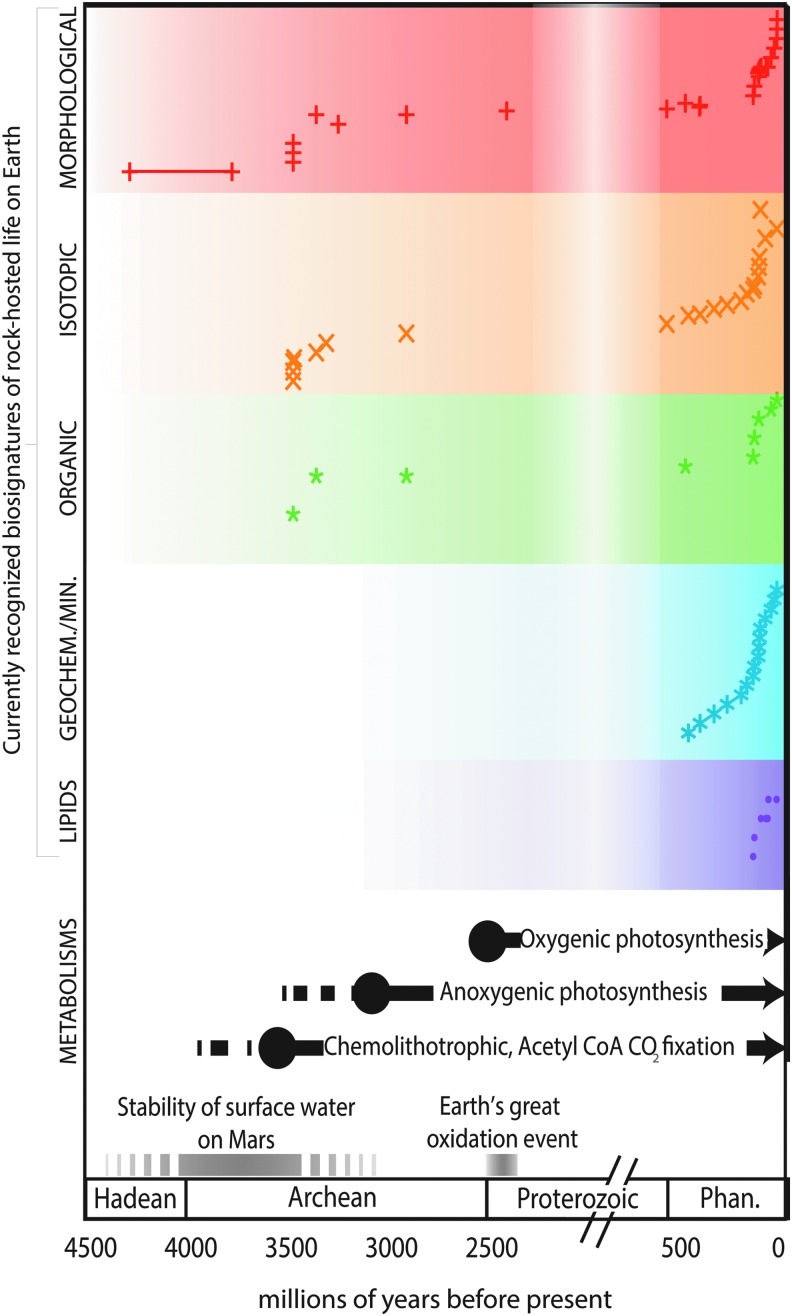
Present understanding of rock-hosted life over time. The currently recognized biosignatures of rock-hosted life from Table 1 are plotted as a function of time along with the timing of development of microbial metabolisms from molecular clock techniques, as discussed in the text. Earth's geologic timescale, Earth's oxidation, and the era of surface stability of water on Mars are also shown.

Examination of fossil evidence for life on Earth prior to ∼2 Ga is hindered by the fact that single-cell prokaryotes typically do not produce inorganic cellular components and Archean rocks have been subjected to metamorphic conditions capable of completely erasing the organic microscopic cellular remains. For those rare low-metamorphic-grade Archean rocks, molecular biosignatures such as hopanes (Eigenbrode, 2008) and their associated isotopic signatures (Williford *et al.,* 2016) can constrain ancient metabolic processes. Examining examples of fossilized subsurface ecosystems in Phanerozoic rocks, however, provides a bridge between modern-day processes and contestable Archean examples (Table 1; Fig. 4).

**Table 1.** Subsurface Biomarkers Preserved in Geological Record

*Age (Ma)*	*Biosignature type reported*					*Forms*	*Formation type*	*Ref.*
	*Isotope*	*Geochemistry/mineralogy*	*Morphology*	*Organic carbon*	*Lipid*			
0.6			x			filaments	Sea Mounts	1
1			x	x		filaments	Ries Impact Crater	2, 3
2	x	x	x			concretions	Navajo Sandstone	4
3.4–44					x		Cretaceous shale	5, 6
15		x	x			microcolonies	Columbia River Basalt	7
31		x	x	x		ichnofossils	sea floor basalt	8
48			x		x	filaments	Sea Mounts	9, 10
56			x		x	filaments	Sea Mounts	9
60	x	x				concretions	Moeraki Formation	11
81			x		x	filaments	Sea Mounts	9
84	x	x				concretions	Gammon Shale	12
88.5	x	x	x			concretions	Mancos Shale Formation	13
91	x	x	x			concretions	Frontier Formation	13
95	x	x	x			concretions	Frontier Formation	13
92			x	x		ichnofossils	Troodos ophiolite	14, 15
0.115–400	x	x	x	x	x	microcolonies	Fennoscandian shield granite	16–18
120	x	x	x	x	x	microcolonies	Southern Iberia Abyssal Plain	19
152	x	x				concretions	Kimmeridge Clay	20
173 ± 8	x						Fennoscandian shield granite	21
180	x	x				concretions	Upper Lias	22
250	x	x				reduction spheroids	Mercia Mudstone Group	23
315	x	x				concretions	Lower Westphalian coal	24
355 ± 14	x			x	x	filaments	Fennoscandian shield granite	21
358–394	x			x	x		Fennoscandian shield granite	21, 24
385	x	x	x			filaments	Arnstein pillow basalt	25, 26
388			x			filaments	Tynet Burn limestone	27
443	x	x				ichnofossils	Caledonian ophiolite	28
458			x	x		filaments	Lockne Impact Structure	29
551	x		x			concretions	Doushantuo Formation	30
1175		x				reduction spheroids	Bay of Stoer Formation	23
1950	x	x				ichnofossils	Jormua ophiolite complex	31
2400			x			filaments	Ongeluk Formation sea floor basalt	32
2900–3350	x		x	x		ichnofossils	Euro Basalt	33, 34
3240			x			filaments	Sulphur Springs Group	35
3300	x						Barberton Greenstone Belt	36
3460	x						Dresser Formation	37
3465	x		x	x		microcolonies	Apex Chert	38
3465			x			microcolonies	Apex Chert	39
3465	x						Apex Chert	40
3465	x		x			ichnofossils	Hooggenoeg Formation	41, 42
3770–4280			x			filaments	Nuvvuagittuq belt	43

1 Ivarsson *et al.* (2015), ^2^Sapers *et al.* (2014), ^3^Sapers *et al.* (2015), ^4^Loope *et al.* (2010), ^5^Ringelberg *et al.* (1997), ^6^Elliott *et al.* (1999), ^7^McKinley *et al.* (2000), ^8^Cavalazzi *et al.* (2011), ^9^Ivarsson *et al.* (2009), ^10^Ivarsson *et al.* (2012), ^11^Thyne and Boles (1989), ^12^Coleman (1993), ^13^Mcbride *et al.* (2003), ^14^Furnes *et al.* (2001), ^15^Wacey *et al.* (2014), ^16^Pedersen *et al.* (1997), ^17^Drake *et al.* (2015a), ^18^Drake *et al.* (2015b), ^19^Klein *et al.* (2015), ^20^Irwin (1980), ^21^Drake *et al.* (2017a), ^22^Coleman and Raiswell (1995), ^23^Spinks *et al.* (2010), ^24^Curtis *et al.* (1986), ^25^Drake *et al.* (2018), ^25^Eickmann *et al.* (2009), ^26^Peckmann *et al.* (2007), ^27^Trewin and Knoll (1999), ^28^Furnes *et al.* (2002), ^29^Ivarsson *et al.* (2013), ^30^Dong *et al.* (2008), ^31^Furnes *et al.* (2005), ^32^Bengtson *et al.* (2017), ^33^Banerjee *et al.* (2007), ^34^McLoughlin *et al.* (2012), ^35^Rasmussen (2000), ^36^Ohmoto *et al.* (1993), ^37^Shen and Buick (2004), ^38^Schopf *et al.* (2018), ^39^Pinti *et al.* (2009), ^40^Ueno *et al.* (2006), ^41^Banerjee *et al.* (2006), ^42^Furnes *et al.* (2004), ^43^Dodd *et al.* (2017).

### 4.1. Subsurface life biosignatures in hydrothermally altered ultramafic rocks

Magma-poor paleocontinental margins expose large volumes of mantle peridotites to infiltration by seawater (Whitmarsh *et al.,* 2001). Along the Lower Cretaceous Iberian margin, seismic data indicates 25–100% serpentinization-driven alteration of ultramafic crust to depths of 4 km (Dean *et al.,* 2000). The upper ∼1 km is most heavily altered with serpentinized rocks crosscut by calcite-brucite assemblages, for which isotopic data indicate precipitation at temperatures from 25–40°C. The contact zone is hypothesized to represent the deep plumbing of a Cretaceous “Lost City” type hydrothermal system (Kelley *et al.,* 2005, 2015).

Mineralized veins in the contact zone at depths of ∼750 m are significantly enriched in organic carbon. Analysis for biosignatures revealed round to rod-shaped structures, ∼2–200 μm in diameter, which are consistent with the morphologies of microbial colonies. Analyses of these putative fossilized cells with Raman spectroscopy revealed them to be carbon-enriched, with C–H, –CH_2_, and –CH_3_ functional groups. Band positions are consistent with lipids, amino acid side chains of proteins and carbohydrates, and amide I bonds in proteins. Further analysis of lipid biomarkers revealed nonisoprenoidal dialkylglycerol diether lipids of bacterial origin and acyclic glycerol dibiphytanyl glycerol tetraether lipids of archaeal origin. Thus, hydrothermal activity ∼750 m beneath the seafloor at ∼120 Ma sustained very abundant archaeal and bacterial microbial communities, equivalent to ∼10^9^ cells cm^−2^, within fractures leaving behind morphologic fossils, organic carbon, and lipids (Klein *et al.,* 2015).

Fossilized remains of microorganisms have also been described in carbonate or serpentine veins of ∼1 Ma ultramafic peridotite rocks in the Mid-Atlantic Ridge, and characterized by a combination of morphology, chemical composition, and the presence of organic matter, sometimes including specific complex amides usually characteristic of biopolymers (Ménez *et al.,* 2012; Ivarsson *et al.,* 2018). In these systems, complex organics are found in either aragonite veins or within a poorly crystalline mix of serpentine, magnetite, and hydrogarnet, and remnant orthopyroxene with chemical enrichments in Ni, Co, Mo, and Mn (Ménez *et al.,* 2012; Ivarsson *et al.,* 2018) that are different compared to microbial preservation in basalt-hosted systems, which are instead dominated by clay minerals or Fe oxides (see Section 4.2 below).

### 4.2. Fracture-filling fossilized complex subsurface chasmoendolithic and cryptoendolithic communities in igneous rocks and precipitated rocks

Basaltic rocks cored from modern-day continental groundwater circulation sites show that cells are strongly concentrated within clay and oxides assemblages in fractures and pore spaces (*e.g.,* Trias *et al.,* 2017, their Supplementary Fig. 15). Encrustation of biological matter by mineralization is a key means of preserving the structures over geologic time, as cataloged for rocks of multiple ages and types (Hofmann and Farmer, 2000; Hofmann, 2008). Investigations of ancient, now fossilized fracture surfaces in igneous rocks show these are zones of concentration of microbial activity, sometimes including complex communities of organisms with multiple trophic levels, preserved by mineralization.

In seamount basaltic lavas ranging in age from 48 to 81 Ma, the fossilized remains of chasmoendolithic (fracture-dwelling) subsurface microorganisms, that is, coccoidal, filamentous or stromatolitic structures with elevated carbon concentration and organic matter such as lipids and rare chitin, are preserved in mixtures of clay, Fe oxides, and Mn oxides and carbonate and gypsum veins (Ivarsson and Holm, 2008; Ivarsson *et al.,* 2009, 2012; Bengtson *et al.,* 2014). The microstromatolitic structures found at 68–153 m below the seafloor within the fractured basalt are interpreted as the result of Fe- and Mn-oxidizing bacteria, and based on mineral succession, appear to be the initial colonizers of subseafloor basalt (Bengtson *et al.,* 2014; Ivarsson *et al.,* 2015). These rocks are interpreted to preserve a syntrophic community of chemolithoautotrophs, hyphae-forming fungi, and microstromatolitic *Frutexites.* Most filamentous and coccoidal fossils have so far been interpreted on morphological characteristics and rare chitin as fungal hyphae and yeast growth stages, respectively (Ivarsson *et al.,* 2012, 2015). This is probably not due to a dominance of fungi in subseafloor crust but instead due to fungi being more easily recognized than prokaryotic fossils. Microstromatolites and associated single-celled features with morphologies comparable to S-cycling archaea like *Pyrodictium* species suggest prokaryotic remains are present as well (Bengtson *et al.,* 2014; Ivarsson *et al.,* 2015). The organic micron-sized coccoidal shapes occur in concentrations equivalent to ∼10^7^ cells cm^−2^ on the vein walls with vein-containing tubular ichnofossils (see Section 4.3 below) and with saline fluid inclusions recording entrapment temperatures of ∼130°C.

As a second example, in continental flood basalts of Miocene age (17–6 Ma) in Oregon, secondary minerals formed within and near fractures preserve ∼1 μm sized coccoidal and rod-shaped microstructures and framboidal pyrite associated with kerogen (McKinley *et al.,* 2000) in an aquifer where the present-day microbial communities are comprised of sulfate-reducing bacteria (Baker *et al.,* 2003). Iron oxyhydroxides, smectites, zeolites, and silica within fractures and smectite veins were investigated because they contained framboidal pyrite, and the cell-like microstructures were discovered.

As a third example, fossilized remains of biofilms have also been found preserved within calcite-filled fractures of 1800 Ma granite at a depth of 200 m in the Fennoscandian shield using transmission electron microscopy (Pedersen *et al.,* 1997). Stable isotope, fluid inclusion, and fission track analyses constrain the age of occupation and entrapment between 115 ka and 400–300 Ma. More recently, Drake *et al.* (2017a) have discovered fossil biofilms preserved in calcite veins dated at 355 ± 14 Ma that occur at 300 m depth in a similar Swedish granite and core lipids indicative of sulfate-reducing bacteria extracted from calcite veins from 80 to 920 m depth in Swedish granite and that are 358–394 million years in age (Drake *et al.,* 2018). In the same granitic rocks, coupled bacterial sulfate reduction–anaerobic CH_4_ oxidation paleoactivity is recorded by the −125‰ to +36.5‰ V-PDB δ^13^C values and diagnostic lipid biomarkers preserved in vein-filling calcite (Drake *et al.,* 2015a) that formed at temperatures <50°C and the −54‰ to +132‰ V-CDT δ^34^S values in pyrite lining open fractures (Drake *et al.,* 2015b, 2018) over a depth range of 200–750 m. These paleobiosignatures are consistent with the present-day observations of a coupled bacterial sulfate reduction–anaerobic CH_4_ oxidation zone over similar depth ranges in fracture water from the granite, though in one instance the sulfate-rich zone is above the methane-rich zone (Pedersen *et al.,* 2014), and in the other instance the opposite is true (Hallbeck and Pedersen, 2012).

Excellent preservation of fossilized fungal mycelia have also been reported from granites of the Fennoscandian shield with diagnostic morphologies like anastomosis between branches (Ivarsson *et al.,* 2013). Drake *et al.* (2017b) have reported a fossilized anaerobic fungi–sulfate reducing bacteria consortium from a 740 m deep fracture in granite located at the Laxemar site, Sweden. In particular, fossils of filamentous microorganisms of fungi are within impact-induced fractures in granite from the 89 Ma Dellen impact and 458 Ma Lockne impact (Ivarsson *et al.,* 2013). Both sites are related to the subsequent hydrothermally formed mineralization, indicating that impact-generated habitats in igneous rock can be favorable for microbial colonization and preservation.

As a fourth example, some organisms, known as “autoendoliths,” play a more active role in the formation of rock edifices, whose precipitation can result directly from microbial metabolism and encapsulate the responsible microbial constituents (Marlow *et al.,* 2015). For example, anaerobic methanotrophs oxidize methane, increase alkalinity, and produce bicarbonate that precipitates as carbonate rock at methane seeps (Peckmann *et al.,* 2001). Metabolic activity continues from within the rock (Marlow *et al.,* 2014b), and biosignatures of the entombed organisms can persist for hundreds of millions of years (Peckmann and Thiel, 2004).

Other examples of fracture-filling filamentous fabrics of rock-hosted life include those preserved in chalcedony and/or zeolite in tens of terrestrial volcanic rocks ranging in age from Tertiary to Mesoproterozoic (Hofmann and Farmer, 2000); filamentous fabrics of what are interpreted as Fe-oxidizing chemotrophic bacteria of Devonian age in calcite veins cross-cutting lacustrine sedimentary rock (Trewin and Knoll, 1999); complex mineralized filamentous structures in Devonian-age pillow basalt (Peckmann *et al.,* 2007; Eickmann *et al.,* 2009); and fungi-like mycelial fossils in vesicles and fractures of 2.4 Ga basalt in South Africa (Bengtson *et al.,* 2017). The interpretations that these fossils represent subsurface prokaryotes and fungi are consistent with observations of the present-day subsurface biosphere (see Section 3.1). Nonetheless, as the record is pushed backward, Earth's overprinting processes demand more sophisticated high-resolution analyses for biogenicity determination (see also Section 4.6).

### 4.3. Microbial trace fossils in recent and ancient glass

Complex, tubular structures that are sometimes found emanating from alteration mineral–filled fractures in basalts, basaltic glass, or impact glass may in some cases be trace fossils of microbial origin, called ichnofossils. These structures were first reported in Pleistocene volcanic glass in Iceland (Thorseth *et al.,* 1992) and have since been documented globally in young seafloor pillow basalt glass (Thorseth *et al.,* 1995; Furnes *et al.,* 1996; Fisk *et al.,* 1998; Fisk and McLoughlin, 2013) and in pillow basalt of ancient ophiolites and greenstone belts dating back 3.5 Ga (Furnes *et al.,* 2008). The mechanisms of formation of these features combine dissolution of glass with leaching of cations and formation of clay minerals, Fe and Mn silicates, and Fe and Ti oxides (Staudigel *et al.,* 2008).

In modern rocks, basaltic glass samples from 1.3–1.8 km depth from Hawaii Scientific Drilling Program core that were examined with Raman, deep UV fluorescence, and 16S rRNA sequencing show microorganisms are present in clays at the dissolution boundary with the glass near microtubular structures (Fisk *et al.,* 2003). In modern seafloor basalts on the Mohns spreading ridge of the Norwegian Sea, tubular dissolution structures originate from the palagonite-glass interfaces, and evidence for bacterial processing includes the characteristic rounded and elongated, microbial-sized, 0.5–2 μm, pores, enrichments of Mn on the rims of coccoid-shaped structures with elevated concentrations of C, N, and organic carbon with a depleted isotopic signature (Kruber *et al.,* 2008; McLoughlin *et al.,* 2011).

Determining biotic versus abiotic origin in fossil rocks requires careful observations of textural subtleties and paired morphological-chemical criteria (*e.g.,* McLoughlin and Grosch, 2015). As with many exothermic inorganic chemical processes that are exploited by chemolithoautotrophic microorganisms, distinguishing structure formed by abiotic reactions from biologically mediated ichnofossils is challenging (Knowles *et al.,* 2012; Grosch and McLoughlin, 2014; Wacey *et al.,* 2017). We describe two of the best ancient examples involving rock-hosted microbial life associated with glass dissolution.

Ichnofossils like the modern examples are found in volcanic glass of the 92 Ma Troodos ophiolite (Furnes *et al.,* 2001). Tubular dissolution structures possess 3-D spiral morphologies with organic carbon and nitrogen enriched linings (Wacey *et al.,* 2014). Careful analyses of the relationship between infilling clay minerals and organics shows that the organics formed first by microbial extracellular materials and were then mineralized by clays.

In the 14 Ma Ries impact crater, microtubules related to microbial life are found in the impact-generated glass within heavily altered zones with clay minerals and Fe oxides. The carbon-bearing materials have C-H_*x*_ and amide I and II absorption bands from organic materials, not observed in tubule-free areas (Sapers *et al.,* 2014). Further analysis with Raman spectroscopy showed quinoic compounds, and STXM coupled with NEXAFS showed Fe redox patterns in these areas consistent with microbially mediated dissimilatory Fe reduction (Sapers *et al.,* 2015).

### 4.4. Lipid biomarkers of rock-hosted life

Neutral lipid biomarkers have been widely utilized as a biomarker of terrestrial life in sedimentary and petroleum deposits (see review by Brocks and Summons, 2005), and their application to Archaean marine sediments had a significant impact on the understanding of the evolution of prokaryotes and eukaryotes (*e.g.,* Brocks *et al.,* 1999). However, aliphatic and polycyclic lipids in the metamorphosed Archean sediments containing polyaromatic hydrocarbons were later shown to be drilling contamination (French *et al.,* 2015). Unmentioned, and unresolved, is whether some of the low concentrations of bacterial lipids and archaeal isoprenoids found in the rock matrix could in fact have originated from extant and extinct subsurface microorganisms colonizing the rock mass over billions of years.

Analyses of lipid biomarkers in modern marine sediments under anoxic conditions document how rapidly the lipid biomarkers of marine planktonic biomass, both eukaryotic and prokaryotic, are quickly replaced within the water column and the surface seafloor sediment by the bacterial lipids of the subseafloor biosphere (Schubotz *et al.,* 2009). The archaeal lipid half-lives appear to be longer on the order of hundreds of thousands of years (Xie *et al.,* 2012). A geological test for both the age and preservation of subsurface bacterial lipids has been documented in Cretaceous-age marine sediments that were intruded by mafic dikes 3.4 million years ago (Table 1; Fig. 4). Profiles of the phospholipids (active bacteria) and glycolipids (extinct bacteria) both stratigraphically and as a function of distance from the intrusion indicate that the glycolipids result from the decay of phospholipids of subsurface bacteria. These lipids from subsurface bacteria predate the 3.4 Ma intrusion and postdate the 44 Ma age of burial sterilization (*T*_max_ = 125°C) of the marine shale (Ringelberg *et al.,* 1997; Elliott *et al.,* 1999). This persistence indicates that study of lipid biomarkers of rock-hosted life warrants considerably more attention as the microbial record of ancient terrestrial rocks is interrogated.

### 4.5. Putative Archean subsurface versus surface microbial biosignatures

The earliest, commonly agreed upon, preserved microbial structures are in the 3.4–3.5 Ga rock units of the Pilbara Craton, Australia. Some units contain microfossils and laminated sedimentary structures consistent with stromatolites and contain contentious carbonaceous biosignatures (*e.g.,* Walter *et al.,* 1980; Buick *et al.,* 1981; Schopf, 1993; Brasier *et al.,* 2006; Noffke *et al.,* 2013) that are suggestive of photosynthetic microorganisms at this time, though their biogenicity has been questioned (*e.g.,* Buick *et al.,* 1981; Lowe, 1994; McLoughlin *et al.,* 2008).

The 3.46 Ga Apex chert represents hydrothermal dikes with silica-mineralization containing kerogenous microfossils involved in subsurface cycling of CH_4_ (Schopf *et al.,* 2018) and/or organic matter formed by abiogenic mechanisms, for example, Fischer-Tropsch-type synthesis or remobilization of other organics (Brasier *et al.,* 2002, 2005, 2006; García-Ruiz *et al.,* 2003). Other workers have suggested the −56‰ δ^13^C V-PDB value of CH_4_ trapped in fluid inclusions of the same veins could be the earliest evidence of methanogenesis in the subsurface (Ueno *et al.,* 2006).

Sulfur isotope fractionation between sulfides and barite in sediments and crosscutting veins (Shen *et al.,* 2009) and within seafloor basalt and komatiite (Aoyama and Ueno, 2018) of the Dresser Formation suggest that sulfate-reducing bacteria were also present and metabolically active at the near surface and subsurface by ∼3.46 Ga. Microfossils preserved in chertified Strelley Pool arenite and pyrite with negative δ^34^S V-CDT values also suggest the presence of subsurface sulfate-reducing bacteria at 3.43 Ga (Brasier *et al.,* 2015). Pyritic filaments preserved in the 3.24 Ga deep sea volcanogenic massive sulfide deposits within the Sulphur Springs Group provide the most convincing evidence of early life in the form of thermophilic, chemotrophic prokaryotes living in hydrothermal systems beneath the seafloor (Rasmussen, 2000).

Putative microfossils in the form of mineralized tubular features in basaltic glass from the 3.47–3.46 Ga upper Hooggenoeg Formation of the Barberton Greenstone Belt, containing isotopically light carbonate, have been proposed as subsurface biosphere fossils (Furnes *et al.,* 2004; Banerjee *et al.,* 2006). Titanite mineralized microtubules in 3.35 Ga basaltic glass with a minimum age of 2.9 Ga have also been suggested to represent evidence of biological processing (Banerjee *et al.,* 2007). Very negative δ^34^S V-CDT values from pyrite within these microtubules suggest that they were formed by microbial sulfate reduction (McLoughlin *et al.,* 2012). More recently, however, Grosch and McLoughlin (2014) disputed the biogenicity of the microtubule textures suggesting they represent contact metamorphic textures associated with postdepositional intrusions (Table 1; Fig. 3).

Even more controversial are the earliest traces of life from 3.95–3.75 Ga amphibolite-grade metamorphic rocks in Greenland and Labrador in the form of “biogenic graphite” (Mojzsis *et al.,* 1996; McKeegan *et al.,* 2007; Tashiro *et al.,* 2017) and a single graphite inclusion in 4.1 Ga zircons from Jack Hills, Australia (Bell *et al.,* 2015) that yield negative δ^13^C V-PDB values comparable to those of modern life. Determining whether they represent primary organic matter versus secondary organic matter formed during later metamorphism is challenging (Papineau *et al.,* 2011), and isotopic values do not determine whether they formed by biological fractionation or abiotic processes (van Zuilen *et al.,* 2003; Sherwood Lollar *et al.,* 2006), let alone whether they represent “chemofossils” of phototrophic or subsurface chemoautotrophic microbial biomass. Other evidence for Archean life is based upon textural evidence such as putative centimeter-scale stromatolites in 3.7 Ga metacarbonates in Greenland (Nutman *et al.,* 2016) and tens-of-micron-scale hematite filaments in 4.2 Ga metasedimentary rock in Quebec (Dodd *et al.,* 2017). The former features, however, were recently refuted as true stromatolites (Allwood *et al.,* 2018).

In summary, the paucity of low metamorphic grade Archaean rock record hampers our ability to identify unambiguous biosignatures older than ∼3 Ga, which is approximately the time frame at which Mars' broad-scale habitability began to decline. Putative morphological biogenic structures, combined with C and S isotopic evidence, have been preserved in volcanics, dikes and quartzites that are consistent with subsurface life (methanogenesis and sulfate reduction) while those found in marine sediments are suggestive of photosynthetic life. Noteworthy is a quote from the work of Brasier *et al.* (2015), as follows: “Why are few cellular fossils found in rocks before 2.5 Ga? For decades, the main search image has been cyanobacteria-like assemblages as silicified algal mats and stromatolites. Have we been looking for fossils in the wrong places?” (Brasier *et al.,* 2015). In light of new insights on the magnitude of the rock-hosted biosphere on Earth, it seems clear that while we were not looking in the wrong places, the taphonomic windows and environmental settings investigated for the biomarkers of ancient life should be expanded. The same can be said, therefore, for the >50% of the surface of Mars that is older than ∼3 Ga. As it is unmetamorphosed relative to Earth, it represents a particularly compelling exploration frontier (see Section 5).

### 4.6. The footprint of fossil rock-hosted life

The confirmation of biosignatures often takes place at the micrometer scale by a suite of integrated techniques described in the examples above. Nevertheless, the “footprint” of fossil rock-hosted life can far exceed this scale as the mineralogical, chemical, and isotopic signatures for the presence of life can often be observed in bulk rock samples, sometimes over enormous volumes ranging from millimeters to kilometers. Indeed, the impact of rock-hosted life on biogeochemical cycling on Earth is significant and a current major topic of research (Colwell and D'Hondt, 2013).

**FIG. 5.**
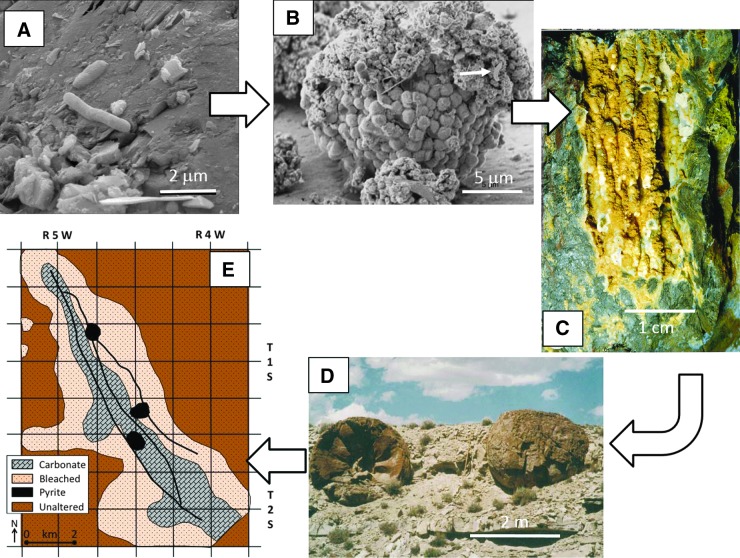
Increasing scale of metabolic footprint of subsurface life. (**A**) Single microbial cells attached to clay minerals of a 2.8 km deep fracture zone (Wanger *et al.,* 2006). (**B**) Framboidal pyrite sack with organic mineralization from 1.5 km deep borehole. White arrow points to single bacterial cell (Maclean *et al.,* 2008). (**C**) Centimeter-scale “Pseudostalactite” of quartz and goethite cemented by biogenic filaments occurring in Tertiary volcanic rocks in California (Hofmann and Farmer, 2000). (**D**) Ferroan carbonate septarian concretions from 88.5 Ma in the Ferron Sandstone Member of the Mancos Shale Formation in Utah that are 1–4 m in diameter (McBride *et al.,* 2003). (**E**) Surface diagenetic alteration zones and traces of pre-Permian faults over Velma field, Stephens County, Oklahoma, 1 mile scale bar (Al-Shaieb *et al.,* 1994).

An example of just how large this footprint can be is the magnetic anomalies on the scale of tens of kilometers detected around oil fields by aeromagnetic surveys (Fig. 5). These anomalies are characteristic of freshwater oil reservoirs and result from the oxidation of hydrocarbon coupled to the reduction of Fe(III) in the sediment by Fe(III)-reducing bacteria producing fine-grained magnetite that records the ambient magnetic field at the time of crystallization over the geological life span of the oil reservoir (Liu *et al.,* 2004; Schumacher, 1996).

An example more relevant to planetary sciences is the footprint left behind by the subsurface microbial processes in pore waters in sediments that successively consume organic matter and give mineralogically, chemically, and isotopically characteristic products that are much larger than the microorganisms forming them. For example, the association of pyrite with nonferroan calcite with δ^13^C values of approximately −20‰ V-PDB is a clear indication of the former presence of sulfate-reducing bacteria, as has been shown in modern-day deposits (Coleman *et al.,* 1993). The likely δ^13^C value for each of the processes, which may vary from −20‰ to +15‰ V-PDB, is summarized in Fig. 1 of Coleman (1985). Any part of a sedimentary succession might have evidence of one process only, and this results from the rate of burial of the sediment (or changes in rate), which controls in which zone the organic matter resides for the longest time (Irwin *et al.,* 1977). The characterization is sufficiently specific that contributions from different processes can be identified in a single mineral, for example, Mn reduction, Fe reduction, and methanogenesis in siderite (Fe carbonate) minerals in 315 Ma sediments (Curtis *et al.,* 1986). These mineral biosignatures occur as intergranular pore-filling cements in many sediment rock types ranging from shale to coarse sandstone (Curtis, 1977). However, the most spectacular occurrences are large spherical or subspherical nodular concretions ranging in size from a few millimeters up to 2 m diameter (Thyne and Boles, 1989). Their visibility makes them excellent pathfinders for other more detailed analyses to confirm their origin, and they can preserve biosignatures for more than 550 million years (Dong *et al.,* 2008).

Other examples of carbonate/oxide concretions produced by anaerobic and micro-aerophilic subsurface bacteria are found in ancient sandstones at scales of up to meters (Coleman, 1993; Abdel-Wahab and McBride, 2001; McBride *et al.,* 2003). These sandstone concretions form as a result of microbially mediated redox reactions occurring during fluid flow long after deposition of the sediment. Meter-sized Fe(II)-rich carbonate/iron oxide concretions (Fig. 4) are found in Jurassic sandstone deposits of southwest Colorado that were formed at hundreds of meters' depth between 2 and 0.5 Ma as the Colorado River Basin was uplifted (McBride *et al.,* 2003; Loope *et al.,* 2010). Similar-sized ferroan calcite and siderite concretions occur in Late Paleocene/Early Eocene Wasatch Group sandstones, and siderite nodule-bearing cores from the formation (Lorenz *et al.,* 1996) yielded thermophilic Fe(III)-reducing bacteria that were capable of producing prodigious quantities of siderite (Roh *et al.,* 2002). In subaqueous systems unconstrained by rock matrix, authigenic carbonate mounds at CH_4_ and hydrocarbon seeps, formed from carbon mobilized by methane- and alkane-oxidizing microorganisms (Greinert *et al.,* 2001; Formolo *et al.,* 2004; Ussler and Paull, 2008), can be hundreds of meters tall and more than a kilometer wide (Klaucke *et al.,* 2008).

At smaller scales but still larger than individual microorganisms, filamentous bacteria often form mats centimeters in size that later are silicified into stalactite-like cavity-filling textures (Hofmann and Farmer, 2000). Smaller still are framboidal pyrites generated by sulfate-reducing bacteria in sizes ranging up to tens of micrometers in diameter (Popa *et al.,* 2004; Maclean *et al.,* 2008). Framboidal pyrites of similar size and texture are seen in Archean sedimentary deposits (*e.g.,* Guy *et al.,* 2012). However, framboidal pyrite alone is not an infallible biosignature, as pyrite with similar microcrystalline textures can be produced abiotically in the laboratory if the solutions are extremely supersaturated with respect to pyrite and/or the temperatures are greater than 60°C (Ohfuji and Rickard, 2005) and occur naturally in ore deposits formed at 150°C to 320°C (Halbach *et al.,* 1993), temperatures well above the limit of hyperthermophiles. Nevertheless, true framboidal pyrites are widely associated with microbial sulfate reduction and can act as a valuable pathfinder so that other characterizations can be performed.

Reduction spheroids were formerly believed to be created by detrital organic matter abiotically reducing Fe(III) minerals to Fe(II) minerals in red beds, but additional mechanisms, such as radiolysis and subsurface bacteria, have been advanced to explain their occurrence (Keller, 1929; Hofmann, 1990, 1991, 2008). Reduction spheroids that range in diameter from millimeters to decimeters with a core enriched in uranium and vanadium have been found throughout the geological record reaching back to the oldest red beds in the Mesoproterozoic and are believed to record the activity of subsurface microorganisms often pre-dating peak metamorphism and deformation (Spinks *et al.,* 2010).

Thus, the footprint of subsurface metabolic activity can greatly exceed the organic content of the microorganisms responsible. To quantify some of the examples above, 1–20 cells occurring within a cluster or patch in the rock would be comprised of only 2 × 10^−15^ to 4 × 10^−14^ mol of organic carbon in a 10^−11^ cm^3^ volume. Microbially generated framboidal pyrite ranges in size and mass up to ∼10^−9^ cm^3^ of 53 wt % S, representing ∼10^−10^ mol e^-^ of sulfate reduction. Reduction spheroids are 10^−3^ to 10^3^ cm^3^ volumes, depleted in Fe(III) by 1–3 wt % Fe_2_O_3_ compared to the rock host (Hofmann, 1990), and represent 10^−6^ to 5 mol e^-^ of Fe(III) reduction. Carbonate concretions attain volumes of 10^3^ to 10^6^ cm^3^, enriched in carbonate by as much as 50–70 wt % (Coleman and Raiswell, 1995), and represent 10 to 10^4^ mol e^-^ from organic carbon oxidation although isotopic profiles suggest that some of the carbonate volume could accrue from increased alkalinity due to anaerobic respiration (McBride *et al.,* 2003). These estimates correspond to a total metabolic conversion of ∼1 (reduction spheroids) to 100 (pyrite framboids) mol e^-^ L^−1^ over timescales of millions of years (Thyne and Boles, 1989; Abdel-Wahab and McBride, 2001; Mcbride *et al.,* 2003). Such rates are consistent with the metabolic rates reported for near-shore, deep subseafloor sediment microbial communities (Orcutt *et al.,* 2013), and the size of these mineral footprints reflects the environmental stability of the subsurface environment over geological time intervals.

## 5. An Exploration Strategy for Past Rock-Hosted Life Biosignatures on Mars

### 5.1. Lessons from Earth

Earth's crust has harbored and preserved subsurface life since at least 3.2 Ga, possibly 3.45 Ga, and the fossil remains are preserved in rock with low metamorphic grade, which is promising for tracking the terrestrial fossil record as well as searching for fossils in similar-age rocks from Mars. The types of biosignatures of rock-hosted life include morphologic structures, organic carbon, spatial patterns in geochemistry, gases, minerals, and their isotopic signatures. When available in concert they can distinguish rock-hosted life from abiotic footprints (*e.g.,* McLoughlin *et al.,* 2011). The criteria for recognizing such life are not fundamentally different from those articulated for more “classic” near-surface sedimentary environments (Summons *et al.,* 2011). As summarized by Grosch *et al.* (2014), textural, chemical, and isotopic information (about both reservoir composition and fractionation patterns) is required, initially at submillimeter scale and then micrometer scale with NanoSims, FIB-TEM, and X-ray synchrotron-based studies. The nature of the rock types that warrant investigation for fossil rock-hosted life and the methods for finding and then characterizing the most promising samples are, however, different. Crystalline igneous rocks altered by groundwater and impact-altered rocks are of high priority in the search for rock-hosted life on Mars. Several heuristic principles can be extracted, based on the terrestrial experience in finding and characterizing biosignatures of rock-hosted life, discussed in Sections 3–4. These scale down from the landscape scale to the microscopic scale (Fig. 4).

First, suitable host rock formations must be identified within the environmental parameters that support life. These include zones with a suitable temperature range during water-rock interaction (< ∼120°C) and sufficient permeability for fluid flow or porosity for diffusive transport (can be highly heterogeneous), combined with redox couples that yield sufficient energy to provide adequate power for sustaining significant biomass concentrations. Many martian rock formations may be suitable (see Section 2). Even rocks identified with higher-temperature water-rock alteration are of interest because there will exist some contact zone or gradient where the higher-temperature waters cool toward Mars surface ambient. Rocks should not have excessive overprinting by later chemical or thermal processes, which might obfuscate or destroy the interpretation of the origin of the rock-hosted life biosignatures. However, even rocks with low-grade hydrothermal or metamorphic overprinting have yielded subsurface biosignatures on Earth, although the duration of such modifications is an important consideration. Ancient martian rocks are generally far less metamorphosed than ancient terrestrial rocks due to the absence of plate tectonics.

Second, finding specific locales to search for biosignatures relies on seeking interfaces at a variety of spatial scales. Studies of terrestrial rock-hosted life (and ice-hosted life) have revealed there are two types of interfaces conducive to rock-hosted life: zones with redox disequilibria gradients or high-permeability zones of fluid flow. The former provide energy for life, and the latter ensure sufficient delivery of new material to support metabolism and removal of waste products. Fault zones, fractured rock, connected vesicles and voids, and alteration zones are locations where rock-hosted life, present and fossil, is found on Earth. Indeed, the cell count is often at least an order of magnitude higher at the interfaces in comparison to surrounding rock (Fig. 3, Section 3.3). Meter-scale and centimeter-scale analyses of morphology, mineralogy, and chemistry can identify these key interfaces for investigation. Detection of the biosignatures themselves relies on smaller spatial scale (submillimeter) analyses of the patterning in morphology, chemistry, and isotopes.

Third, bulk rock organic carbon content over large spatial scales does not track as a key indicator of the richness of the microbial community. Heterogeneity along interfaces is expected, and most subsurface cell concentrations are clustered rather than diffuse. Based upon a review of terrestrial biomass distribution (see Section 3.3), any search for cell-like materials requires searching rock fracture surfaces for ∼10 cell clusters (> ∼10^3^ cells g^−1^) occupying hundreds of μm^2^ areas or identifying seams with carbonaceous material where cell concentrations can reach 10^9^ cells g^−1^. Thus, the ability to detect 1000 cells g^−1^ at 100 μm spatial sampling may be a rule of thumb for evaluating candidate techniques for *in situ* biosignature prospecting for rock host.

Finally, a crucial lesson from the terrestrial record of fossil rock-hosted life is that the initially detected potential biosignature is more likely to be a suggestive mineralogical, chemical or isotopic composition, possibly in a particular shape or texture, rather than a direct detection of organic carbon enrichment from such life. This is because the products of life are more volumetrically significant than life itself (Section 4.6). Phases that may be metabolic products of rock-hosted life or be by-products of metabolic reactions include sulfide, carbonate, sulfate, oxides as well as gases trapped in fluid inclusions. The ability to interrogate their microscale textures, isotopic signatures, the presence or absence of organic carbon, and trace element patterns all support the ability to identify possible biosignatures that later might be confirmed with still smaller-scale analyses in terrestrial laboratories.

### 5.2. The exploration strategy for Mars

On Mars, rocks preserving ancient subsurface habitats are accessible to surface exploration today. Their interrogation does not require the type of large or complex drilling operations that have been proposed to search for modern deep subsurface life beneath kilometers of rock or ice (Stamenković *et al.,* 2019). The search for paleo-rock-hosted life is considerably more straightforward and can be conducted efficiently at many locations with rovers or other mobile surface explorers. This is because faulting, impacts, and erosion have exposed scarps of rocks from the subsurface and ongoing wind erosion continually renews the upper surface to expose rocks less affected by radiation and oxidation. Crustal loading by emplacement of large volcanic edifices at Tharsis and unloading around large impact basins around Isidis and Argyre have created extensional faults that expose thick strata of crust that once hosted groundwater flow (Ehlmann *et al.,* 2011; Ehlmann and Edwards, 2014). Active erosional processes have exposed hundreds of meters of materials that once hosted aquifers in a form accessible to rovers. Examples include mineralized ridges at centimeter- to kilometer-length-scale, which were conduits of groundwater flow (Thollot *et al.,* 2012; Saper and Mustard, 2013; Siebach and Grotzinger, 2014; Quinn and Ehlmann, 2019). At Gale Crater, erosion rates that refresh the surface and expose materials less affected by radiation are ∼0.75 m My^−1^ (Farley *et al.,* 2014). Impact craters also provide direct exposure of subsurface material by their walls, ejecta, and uplift of materials in the central peak (Cockell and Barlow, 2002; Cockell *et al.,* 2012). The complicating factors affecting preservation of rock-hosted life biosignatures on Earth such as organic matter degradation by modern organisms and overprinting of chemical/mineralogical/isotopic signatures by metamorphic fluids billions of years later are likely absent or reduced in the near subsurface of Mars. Rock-hosted-life biosignatures sealed in reduced mineral phases in the martian subsurface may also be less susceptible to secondary oxidation during uplift and exposure to surface oxidation than surficial porous sediments comprised of oxidized mineral phases.

The exploration strategy for searching for evidence of martian rock-hosted life parallels that employed on Earth, but with the need to narrow progressively the spatial scale of the exploration zone to target efficiently, access, and explore the best sites, given the prevalence of orbital data and—relative to the terrestrial situation—the paucity of opportunities for data collection from landed missions (Table 2). Additionally, many biosignatures are (at present) only detected with advanced laboratory analyses necessitating parsimonious sample selection coupled with acquisition of contextual data for return of those samples with promising preservation of biosignatures for rock-hosted life.

**Table 2.** Steps to Search for Rock-Hosted Life on Mars

*Step*	*Spatial scale*	*Key measurement requirements*
1. Identify rocks with ancient subsurface habitats	<100 m sampling	Ability to identify water-related mineral deposits from orbit and determine stratigraphic context
2. Locate interfaces that represent favorable locations for rock life	Meter- to centimeter-scale	Ability to identify redox and permeability interfaces by identification of distinct lithologic units
3. Search for mineralization from fluid flow at interfaces	Centimeter- and millimeter-scale	Ability to identify silica, carbonate, sulfate, phyllosilicate, and oxides that may mineralize microbial life
4. Search for organics, mineralization, and isotopic anomalies at the interface	<100 μm sampling	Ability to detect organics, chemical, mineralogic, and/or isotopic differences between interface rocks and surrounding rocks indicative of biosignatures
5. Map putative biosignatures in 3-D, tracking chemical and organic variations with texture	<1 μm sampling in 3 dimensions	Ability to identify microbial textures and distinguish biotic and abiotic processes to confirm definitively fossil rock-hosted life

The scaled exploration strategy for rock-hosted life relies on seeking interfaces and boundaries (Table 2). Redox interfaces, indicated by mineralogy with contrasting oxidation states, can be manifest at a range of spatial scales indicating the potential for past thermodynamic disequilibria that drive metabolism. Lithological interfaces that indicate zones of focused fluid flow—fault zones, dikes, fracture networks, and connected vesicles—also are required for exchange of materials with the environment. Because of the importance of subsurface hydrology in establishing and maintaining habitable conditions, reconstructing fluid flow regimes through martian aquifers is a key priority. Volcanic rocks inherit large-scale fractures during cooling. Dike swarms also produce kilometers-scale fracture conduits due to the difference in rock properties at their contacts with bedrock. Sediment compaction and closing of pore space is less pronounced on Mars than it is on Earth due to its lower gravity. Meteorite impacts represent one of many reliable modes of fracturing rock and creating reactive surface area and permeability enhancement—as demonstrated at the Haughton and Chesapeake Bay Impact Structures (Pontefract *et al.,* 2016)—all of which improve habitability prospects (Cockell *et al.,* 2012).

As an example of the exploration strategy for Mars, orbit-based data can identify rocks' lithologies, recording the paleoenvironmental conditions with groundwater flowing through sulfate- and serpentine-containing rocks at Northeast Syrtis Major. The presence of serpentine alongside oxidized sulfur indicates the presence of redox interfaces. Abundant fracturing in the area and the presence of secondary minerals (Quinn and Ehlmann, 2019) suggest lithological interfaces and substantial fluid flow. Calculations of Gibbs free energy suggest that these martian habitats had the necessary energy to support anaerobic oxidation of CH_4_ (Marlow *et al.,* 2014a). Mobile surface explorers with camera and instruments for remote assessment of mineralogy and chemistry can then pinpoint lithologic and mineralogical interfaces such as fractures, redox fronts, and zones of low-temperature aqueous mineralization: for example, crosscutting serpentine veins, serpentine-carbonate contacts, and zones of intense magnetite precipitation to meters then centimeters in scale. Advanced instruments for petrology employed in contact with the rock then examine a variety of initial observables, characteristic of sites hosting signatures of paleo-rock-hosted life, including organics and organic-mineral associations (Tables 1 and 2). In select cases (*e.g.,* complex mineralized filaments, reduction spheroids, concretions or framboidal pyrite), a biosignature may be deemed highly likely, especially if concentrations of organic matter are associated with it. However, the best confirmation of biogenicity would require further higher-resolution laboratory analyses on Earth that include significant sample preparation and nanometer-scale analyses.

In addition to the igneous and sedimentary rocks at Northeast Syrtis and Nili Fossae, igneous and sedimentary rocks altered by groundwater at Valles Marineris (Thollot *et al.,* 2012), sediments in the Terra Sirenum craters (Wray *et al.,* 2011; Ehlmann *et al.,* 2016b), and even Gale Crater present opportunities for searching for subsurface life. Organics have been found in diagenetically altered Gale Crater sediments, though their origin as sedimentary detritus or from later fluids cannot be established from the bulk sample composition reported from the Curiosity rover's instruments (Eigenbrode *et al.,* 2018). As of this writing, the long-lived subsurface habitats on Mars have not yet been targeted in geological or astrobiological investigations of the Mars exploration program, which has instead targeted depositional basins, following an Earth environmental model. As subsurface habitats for rock-hosted life are the most promising sites for preservation of ancient martian life (Section 2), the best prospects for life on our neighboring world await future exploration by *in situ* missions or sample return.

## 6. Summary and Recommendations for Future Directions

A review of the published studies on abundance and diversity of extant terrestrial subsurface life, the diverse environments in which it is found, their fossil remains and biomarkers, and a comparison of the evolution of key metabolic pathways for phototrophic versus chemolithoautotrophic microorganisms provide guidance to the search for biomarkers of subsurface life on Mars. First, the metabolic pathways for microorganisms found in the terrestrial subsurface evolved much earlier in Earth's history than those of surface-dwelling phototrophic microorganisms. Second, time-equivalent environments on Mars were much less stable than on Earth, and martian surface environments were challenged by radiation, aridity, freezing temperatures, and frequent obliquity-driven climate change that reduced the availability of water.

Subsurface environments inhabited by rock-hosted life are common, not rare, on Earth. Rock-hosted life and its preserved remains are found in ultramafic serpentinizing systems, deep groundwater systems, hydrothermal systems, and shallow aquifer and diagenetic environments. Terrestrial subsurface biomass concentration tends to be highest at chemical redox gradients and at permeability interfaces; it does not correlate directly with the abundance of organic carbon. Rock-hosted life does not rely upon metabolizing organic photosynthate supplied by Earth's phototrophic organisms but rather upon subsurface energy sources and fluxes (*e.g.,* water-rock chemical reactions, radiolysis) and the abiotic and biotic recycling of carbon and metabolic waste products. The terrestrial rock record reveals examples of subsurface biomarkers at least back hundreds of millions of years and likely to 3.45 Ga. Several excellent examples of rock-hosted life with high-quality preservation are found in rocks quite different from those traditionally explored for fossils from the photosynthetically supported biosphere.

These findings suggest a well-defined exploration strategy for rock-hosted life on Mars (Table 2):

(1) locate rocks preserving aquifers, that is, the plumbing of hydrothermal and groundwater systems;
(2) then, identify redox interfaces and permeability/porosity boundaries preserved within the rock outcrop;
(3) search for locations where these interfaces exhibit mineralization that may have entombed cells;
(4) at submillimeter scale, interrogate these zones of mineralization for patterns in organic molecule concentration, morphologies suggestive of microbial filaments or cells, changes in isotopic signatures (particularly of C, N, S, and Fe), and associations between these putative biosignatures.

Armed with this strategy, evidence of the biosignatures of rock-hosted life can be found *in situ* on Mars and the best samples identified for return to Earth and further interrogation. The search for rock-hosted life is essential to understanding whether Mars was once inhabited, and the search for life on Mars will only be complete once its subsurface habitats are targeted for exploration.

## Supplementary Material

Supplemental data

## Abbreviations Used

OTUs: operational taxonomic units
SLiMEs: subsurface lithoautotrophic microbial ecosystems
