# Supplementary material for: Paleo-Rock-Hosted Life on Earth and the Search on Mars: A Review and Strategy for Exploration
Source: Astrobiology. 2019 Oct 3;19(10):1230–62. doi: 10.1089/ast.2018.1960 (PMC6786346; doi:10.1089/ast.2018.1960)
Supplement: Supplemental data [file Supp_Fig1.pdf]

Supplementary Data

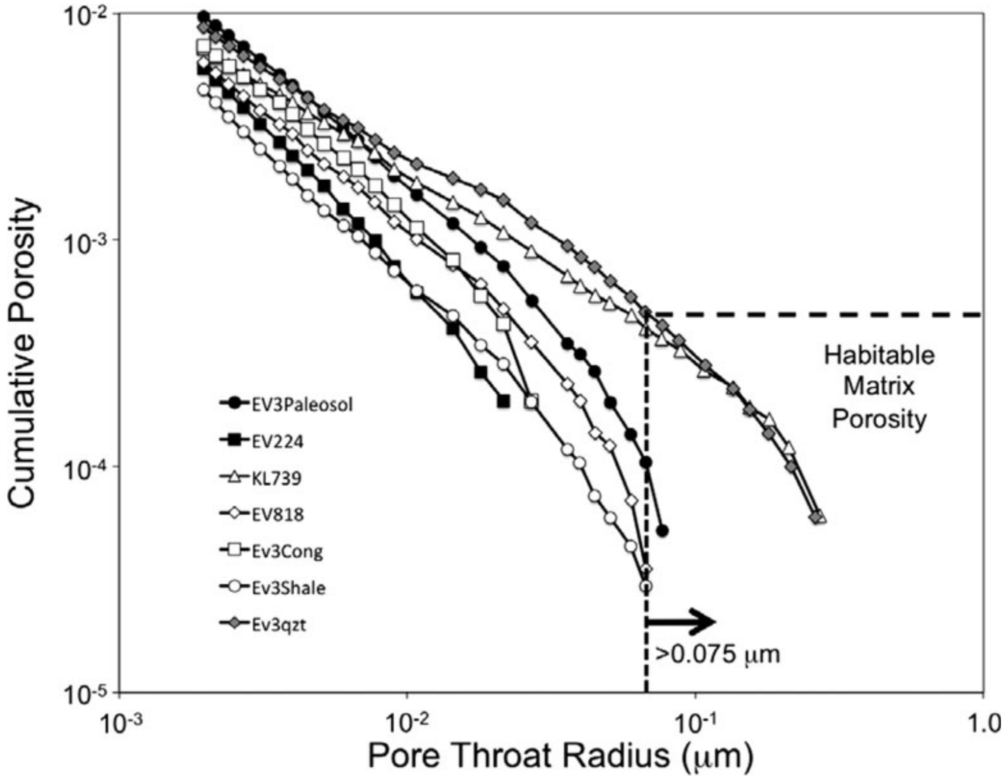

SUPPLEMENTARY FIG. S1. Cumulative porosity in fractional volume versus pore throat radius as measured by Hg porosimetry. Symbols refer to different core samples of sedimentary rock and volcanic rock (KL739) from the Witwatersrand Supergroup. Dashed line demarcates the maximum fractional volume observed for pore diameters greater than 0.15 microns.
